# Analysis of *Acanthamoeba* genotypes from public freshwater sources in Thailand reveals a new genotype, T23 *Acanthamoeba bangkokensis* sp. nov.

**DOI:** 10.1038/s41598-021-96690-0

**Published:** 2021-08-27

**Authors:** Chaturong Putaporntip, Napaporn Kuamsab, Warisa Nuprasert, Rattanaporn Rojrung, Urassaya Pattanawong, Taweesak Tia, Surasuk Yanmanee, Somchai Jongwutiwes

**Affiliations:** Molecular Biology of Malaria and Opportunistic Parasites Research Unit, Department of Parasitology, Faculty of Medicine, Chulalongkorn University, Bangkok, Thailand

**Keywords:** Parasitology, Parasite genetics

## Abstract

A survey of *Acanthamoeba* in 100 public freshwater sources in 28 provinces across Thailand has identified 9 genotypes comprising T2/6, T3-T5, T9, T11, T12, T18 and a novel ‘T23’ among 131 isolates. Sequencing of the near complete 18S rRNA gene of *Acanthamoeba* of all isolates has shown that the most predominant genotype T4 found in 87 isolates (66.4%) contained 4 subtypes, i.e. T4A, T4B, T4C and T4F, while all isolates assigned to genotype T2/6 belonged to subtype B. Among intron-bearing genotypes, most isolates harbouring genotype T3 contained S516 introns, characterised by 3 distinct variants whilst all genotypes T4A and T5 were intronless. Identical 18S rRNA sequences of *Acanthamoeba* were identified across regions of the country and four isolates in this study shared the same sequences with those from remote nations, suggesting that some strains have reproductive success in diverse ecological niche. Nucleotide diversity of genotypes T2/6B, T3, T4, T9 and T11 in this study was significantly less than that among global isolates outside Thailand, implying that limited sequence diversity occurred within local populations. A remarkably higher level of nucleotide diversity in genotype T11 than those of other genotypes (0.041 vs. 0.012–0.024) could be due to cryptic subtypes. Recombination breakpoints have been detected within genotypes and subtypes as well as within isolates despite no evidence for sexual and parasexual cycles in the genus *Acanthamoeba.* Tajima’s *D*, Fu & Li’s D* and F* statistics revealed significantly negative deviation from neutrality across genotypes and subtypes, implying purifying selection in this locus. The 18S rRNA gene of the novel genotype ‘T23’ displayed 7.82% to 28.44% sequence differences in comparison with all known genotypes. Both Bayesian and maximum likelihood phylogenetic trees have placed genotype T23 as sister to the clade comprising genotypes T10, T12 and T14, all of these possess cyst structure belonging to morphological group III. Hence, *Acanthamoeba bangkokensis* sp. nov. is proposed for this novel genotype. It is likely that more genotypes of *Acanthamoeba* remain to be discovered while the evolution of the 18S rRNA gene of this pathogenic-free living amoeba seems to be ongoing.

## Introduction

Since the first discovery of the free-living amoeba in the genus *Acanthamoeba* over nine decades ago^1,2^, it was not until 1974 that the first human case of corneal infection caused by this organism was reported^3^. Subsequently, increasing numbers of patients infected with *Acanthamoeba* have been globally documented. Besides recalcitrant and sight-threatening keratitis which is the most common presentation of acanthamoebiasis, fatal granulomatous amoebic encephalitis and cutaneous infection mostly occurring in immunocompromised hosts are less frequently found^4^. The clinical significance of *Acanthamoeba* has not been limited to its direct pathogenic property per se, several lines of evidence have indicated that a number of microbial endosymbionts have been identified in *Acanthamoeba* isolates in nature. Therefore, *Acanthamoeba* could potentially serve as carriers for a number of human pathogens, such as *Legionella pneumophila*, *Pseudomonas aeruginosa*, *Mycobacterium avium*, *Listeria monocytogenes, Chlamydia* spp. and adenovirus, leading to disease dissemination^4–8^.

*Acanthamoeba* has worldwide distribution and exists in a wide range of environment such as soil, dust, air, freshwater, seawater, sediments and sewage. The life cycle of *Acanthamoeba* contains a replicative trophozoite stage that feeds primarily on a variety of microbes and an environmentally resistant cystic form. Although various species of *Acanthamoeba* have been proposed based on structure of their respective cystic forms and partly from differences in isoenzyme profiles, ambiguity in speciation based on these criteria has compromised their universal application for species assignment^4,9^. Nevertheless, differences in the cyst structure in terms of size and characteristic endocyst and ectocyst of *Acanthamoeba* remain to be useful in morphological classification, characterised by groups I-III^10^. With the advent of a more robust genotype assignment inferred from the nuclear small subunit ribosomal RNA (18S rRNA) sequences^11,12^, worldwide isolates of *Acanthamoeba* can be classified into 22 genotypes, i.e. T1-T22, in which the most predominant genotype isolated from environmental samples and clinical specimens belongs to genotype T4^11–21^. Furthermore, 7 subtypes and 3 supertypes have been identified in genotypes T4 and T2/6, respectively^22^. Meanwhile, not all genotypes of *Acanthamoeba* are known to be incriminated in human infections, suggesting that some genotypes may not possess pathogenic or virulence properties^17,18,23,24^.

Natural freshwater sources are important for agricultural, consumptive and recreational purposes while they can also be reservoirs for disease dissemination to animals and humans. To investigate the extent of genotypic variation of *Acanthamoeba* in natural freshwater sources in Thailand, water samples were collected from 28 provinces across the country. Results reveal wide distribution of *Acanthamoeba* in natural water sources with predominant genotype T4 while a novel genotype has been identified. Further analysis of genetic diversity has suggested that intragenic recombination and purifying selection could contribute to sequence diversity within genotypes and subtypes of *Acanthamoeba*.

## Results

### Distribution of *Acanthamoeba* in water samples

**Figure 1:**
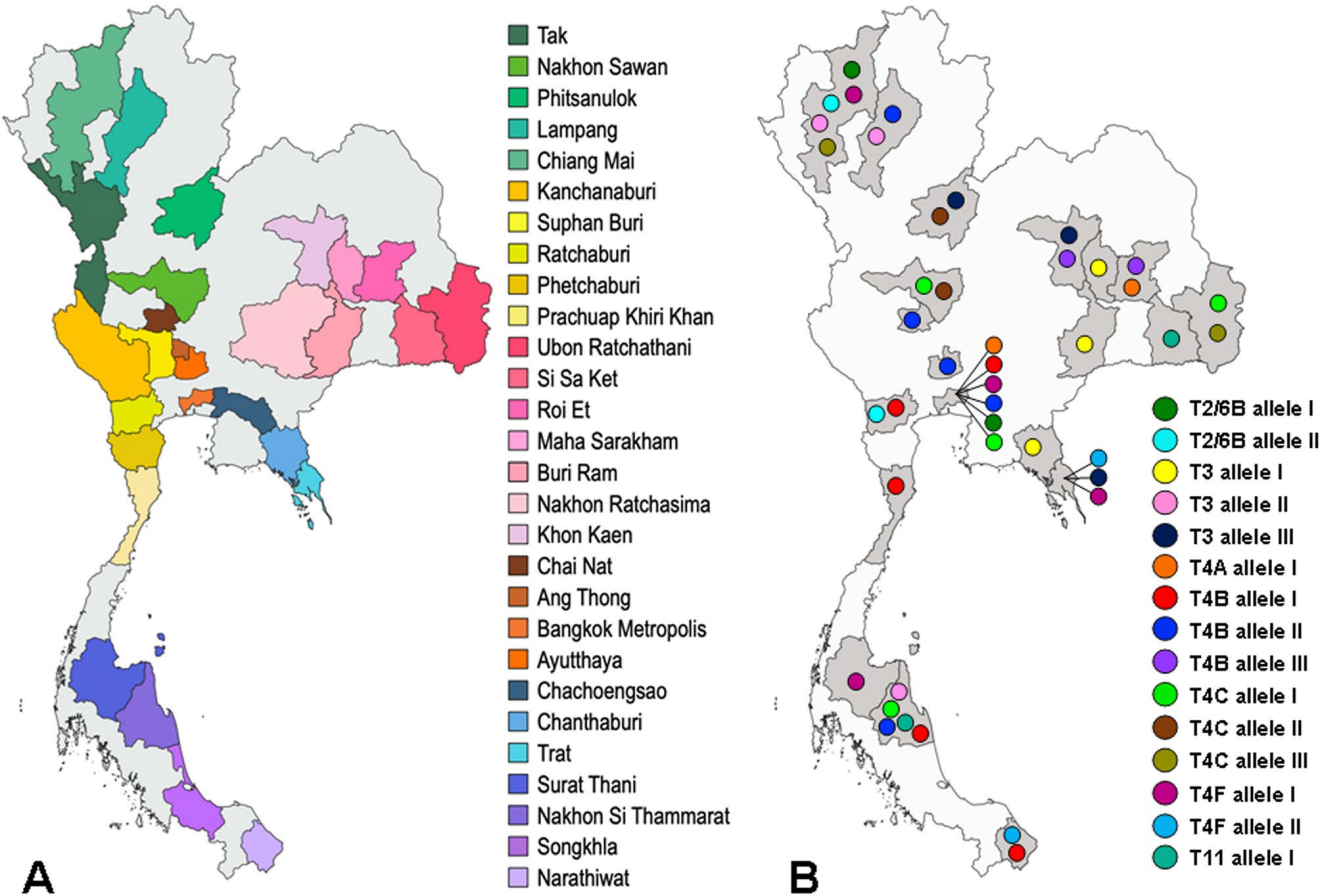
Map of Thailand depicting water sample collection localities (**A**) and corresponding provinces where identical 18S rRNA alleles were detected (**B**) The map is modified from GADM maps and data (https://gadm.org/index.html) under the GADM license version 6.0.

Of 100 water sample collection sites, acanthamoebae were detected from 66 places in 28 provinces across Thailand (Fig. 1 and Supplemental Table S1). Although the proportion of collection sites where *Acanthamoeba* could be isolated was highest in eastern region, followed by western, northeastern, northern, central, and southern regions, respectively, no significant difference between regions was observed (Table 1). The range of acid–base property of water samples was from 6.2 to 8.5 and the temperatures of water by the time of sampling varied from 26 °C to 33 °C. *Acanthamoeba* could be isolated from water with pH ranging from 6.3 to 8.2 and temperatures from 26.5 °C to 33 °C (Supplemental Table S1). The positive rate of *Acanthamoeba* seems not to be associated with pH and temperature of water samples. However, *Acanthamoeba* was more likely to be isolated from still-water than running-water sources (Table 2).

**Table 1:** Distribution of *Acanthamoeba* from water sources by region.

Region	No. provinces	No. sampling sites	No. positive sites (%)*	No. isolates
Northern	4	22	14 (63.64)	22
Central	5	21	13 (61.90)	30
Eastern	3	11	9 (81.82)	12
Northeastern	7	18	12 (66.67)	23
Western	4	8	6 (75.00)	12
Southern	5	20	12 (60.00)	32
Total	28	100	66 (66.00)	131

*No significant differences in the positive rates by pairwise comparison between regions using Fisher exact test (*P* > 0.05).

**Table 2:** Distribution of *Acanthamoeba* in water sources.

Properties of water	No. samples collected	No. positive (%)	*P* value
**pH**			
6.2–6.9	16	7 (43.75)	0.583
7.0–7.9	64	44 (68.75)	
8.0–8.6	20	15 (75.00)	
**Temperature (°C)**			
26–29	28	15 (53.57)	0.157
30–33	72	51 (70.83)	
**Still/Flow**			
Still	82	60 (73.17)	0.0005
Flow	18	6 (33.33)	

Statistics: t-test for pH; Fisher exact test for temperature and still/flow.

### Distribution of *Acanthamoeba* genotypes

**Figure 2:**
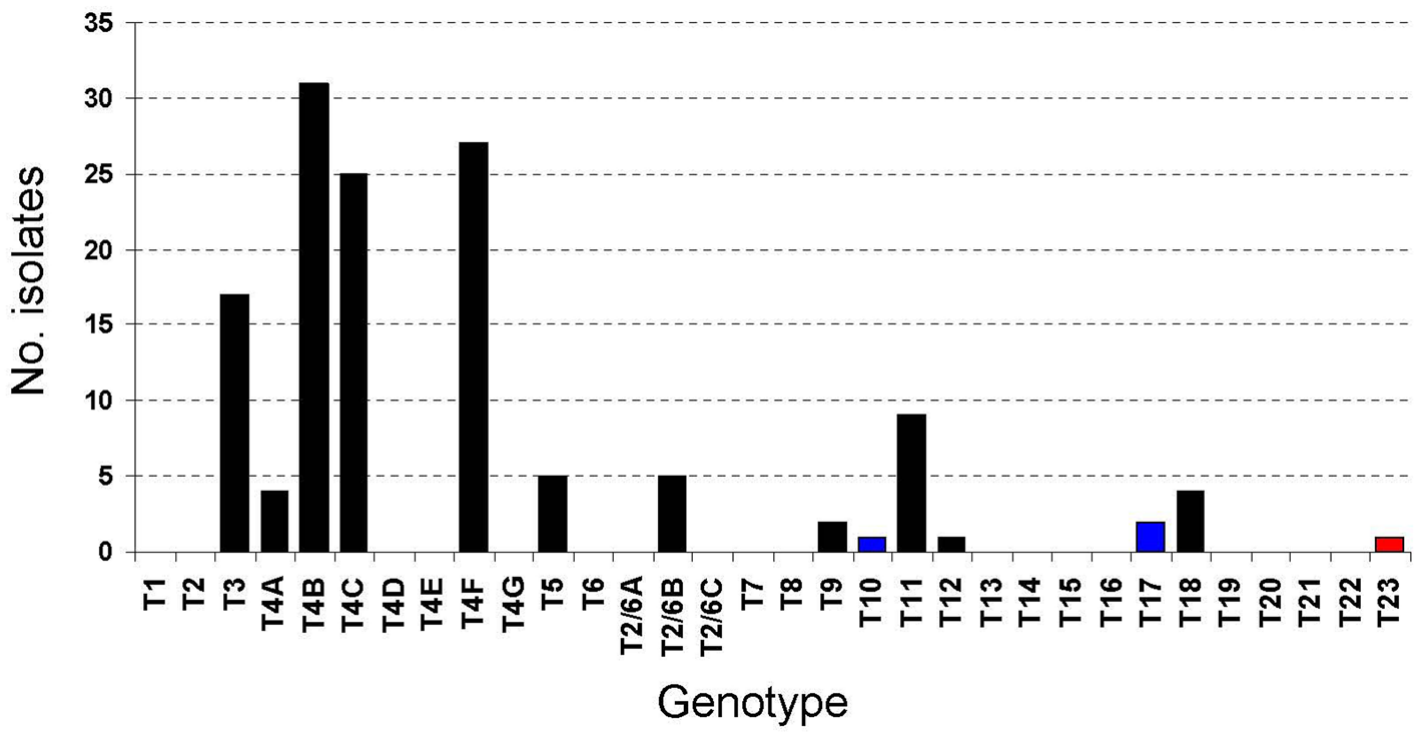
Distribution of genotypes and subtypes of *Acanthamoeba* in Thailand. Black, blue and red bars indicate genotypes in this study, previous survey in Bangkok and the novel genotype, respectively.

Of 66 positive sampling sites, genotypes of *Acanthamoeba* could be assigned for 131 isolates from subculture samples by sequence analysis of the 18S rRNA gene spanning > 2.2 kb. Single genotypes were obtained from 31 sites whilst double, triple, quadruple and quintuple genotypes were found in 16, 10, 7 and 2 sites, respectively (Supplemental Table S1). Six isolates contained two variants of the same genotypes determined by sequencing of secondary PCR products from limiting dilution of primary PCR templates and/or sequencing of recombinant plasmid clones. In total, 137 sequences were obtained. In this survey, 9 genotypes were identified, comprising genotypes T2/6, T3-T5, T9, T11, T12, T18 and a genotype whose sequence differs from other known genotypes. Of these, genotype T4 was most commonly found, occurring in 87 isolates (66.4%). Based on phylogenetic classification proposed by Fuest and colleagues, isolates bearing genotype T4 in this survey could be further assigned to subtypes T4A (n = 4), T4B (n = 33), T4C (n = 27) and T4F (n = 28) whereas all sequences assigned to genotype T2/6 belonged to supertype B (n = 5)^22^ (Fig. 2). With previously reported genotype T10 from a Thai keratitis patient and genotype T17 from water sources in Bangkok, altogether 11 genotypes of *Acanthamoeba* have been identified in Thailand^18^. Meanwhile, the cyst structures based on Pussard and Pond’s classification of 130 isolates, excluding an isolate containing an unknown genotype, were congruent with their genotypes^4^. The most common morphological group of 130 *Acanthamoeba* isolates belonged to group II, accounting for 86.9% while groups I and III could be detected in 7.7% and 5.4%, respectively.

### Sequence diversity within genotypes and subtypes

To analyse the extent of sequence variation in the 18S rRNA gene within genotypes and subtypes of Thai isolates, the corresponding gene sequences from diverse origins available in the GenBank database were retrieved for comparison (Supplemental Table S2). Results revealed that the levels of nucleotide diversity of genotypes T2/6B, T3, T9 and T11 from Thai isolates were significantly less than those from the GenBank database (Table 3). Likewise, the subtypes T4A, T4B, T4C and T4F of Thai isolates had significantly lower levels of nucleotide diversity than those found in more diverse geographic areas. Despite a small number of the T5 genotype sequences among Thai isolates, the level of nucleotide diversity was comparable to those from diverse origins. On the other hand, 12 distinct sequences (alleles) were shared among isolates from two to four different provinces in Thailand (Fig. 1B and Supplemental Table S3). It is noteworthy that these shared alleles belonged to genotype T3 and subtypes T4A, T4B, T4C and T4F, the prevalent genotypes/subtypes of *Acanthamoeba* in this survey. Meanwhile, the predominant alleles in subtypes T4B and T4F in this study possessed identical sequences with isolates from our previous survey in 2009^18^. Furthermore, four alleles in this study were identical with those reported from Scotland (T3 allele I), Russia and South Korea (T3 allele II), Brazil and USA (T4B, allele II) and USA (T4C, allele III)(Supplemental Table S3).

**Table 3:** Nucleotide diversity (π) within genotypes and subtypes of *Acanthamoeba* from Thai and non-Thai origins.

Genotype/Subtype	Total	Thai, n*	π ± S.E		
			All	Non-Thai	Thai
T2	13	0	0.01186 ± 0.00134	–	–
T3	25	17	0.01489 ± 0.00112	0.01793 ± 0.00132	0.00586 ± 0.00052***
T4	313	–	0.018150.00154	–	–
T4A	78	4	0.00972 ± 0.00113	0.00983 ± 0.00113	0.00533 ± 0.00116*
T4B	91	51 (18)	0.00912 ± 0.00104	0.01103 ± 0.00121	0.00648 ± 0.00092**
T4C	53	41 (14)	0.00704 ± 0.00092	0.01189 ± 0.00109	0.00552 ± 0.00094**
T4D	28	0	0.00619 ± 0.00111	–	–
T4E	14	0	0.00597 ± 0.00089	–	–
T4F	40	37 (9)	0.00647 ± 0.00077	0.01281 ± 0.00219	0.00580 ± 0.00070**
T4G	9	0	0.00538 ± 0.00077	–	–
T5	31	5	0.02383 ± 0.00128	0.02483 ± 0.00129	0.02445 ± 0.00226
T2/6B	8	5	0.00661 ± 0.00109	0.00427 ± 0.00108	0.00118 ± 0.00049***
T9	9	5 (3)	0.013000.00129	0.021800.00227	0.005120.00092***
T11	19	11 (2)	0.04083 ± 0.00251	0.04498 ± 0.00264	0.02115 ± 0.00137***
T17	6	5 (5)	0.00852 ± 0.00121	–	0.00656 ± 0.00109
T18	16	4	0.01656 ± 0.00125	0.01251 ± 0.00101*	0.01704 ± 0.00201
T20	12	0	0.01598 ± 0.00146	–	–

Genotypes T1, T2/6A, T2/6C, T6-T8, T10, T12-T14, T16, T19, T21 and T22 are excluded (n < 5 sequences spanning > 2 kb).

*Parentheses denote number of sequences from Thai isolates in GenBank database.

Dash indicates not applicable.

Test of the hypothesis that π_Thai_ = π_non-Thai_; *P < 0.05, **P < 0.005, ***P < 0.0001.

### Intron-containing genotypes

To date, introns have been documented in genotypes T3, T4, T5 and T15, characterised by 4 unique insertion sites, i.e. S516, S943, S956 and S1389^13,25–29^. Sequence analysis has shown that 15 of 17 Thai isolates belonging to genotype T3 possessed an intron insertion at S516 with size variation from 480 to 523 bp. Together with previously reported sequences, a total of 5 intronic variants occurred in genotype T3 in which 4 of these were found in Thailand. Variation in intron sequences were mostly due to insertion or deletion (indel), occurring in the predicted helices P2 and P9, and P1, P2, P5 and P9 loops of the predicted secondary structure^27^ (Supplemental Figs. S1 and S2). Of these, 4 indels were located in short tandem repeat regions: CA repeats in the P2 helix and homopolymeric C, AT and CT repeats in the P9 helix (Supplemental Figs. S1 and S2). The most prevalent intronic variant (n = 11) containing 523 bp shared identical sequence with an isolate from a keratitis patient in Scotland (GenBank accession no. S81337) and 4 of these Thai isolates share identical sequences across the gene. Likewise, a Thai isolate (AcWT29) from northeast region had the same sequence as the isolates from Slovakia and Czech Republic (GenBank accession nos. GQ397468 and GQ905490). Meanwhile, no intron was detected among Thai isolates bearing genotypes T4 (n = 131) and T5 (n = 5) while genotype T15 was not found in this survey.

### Intragenic recombination

**Figure 3:**
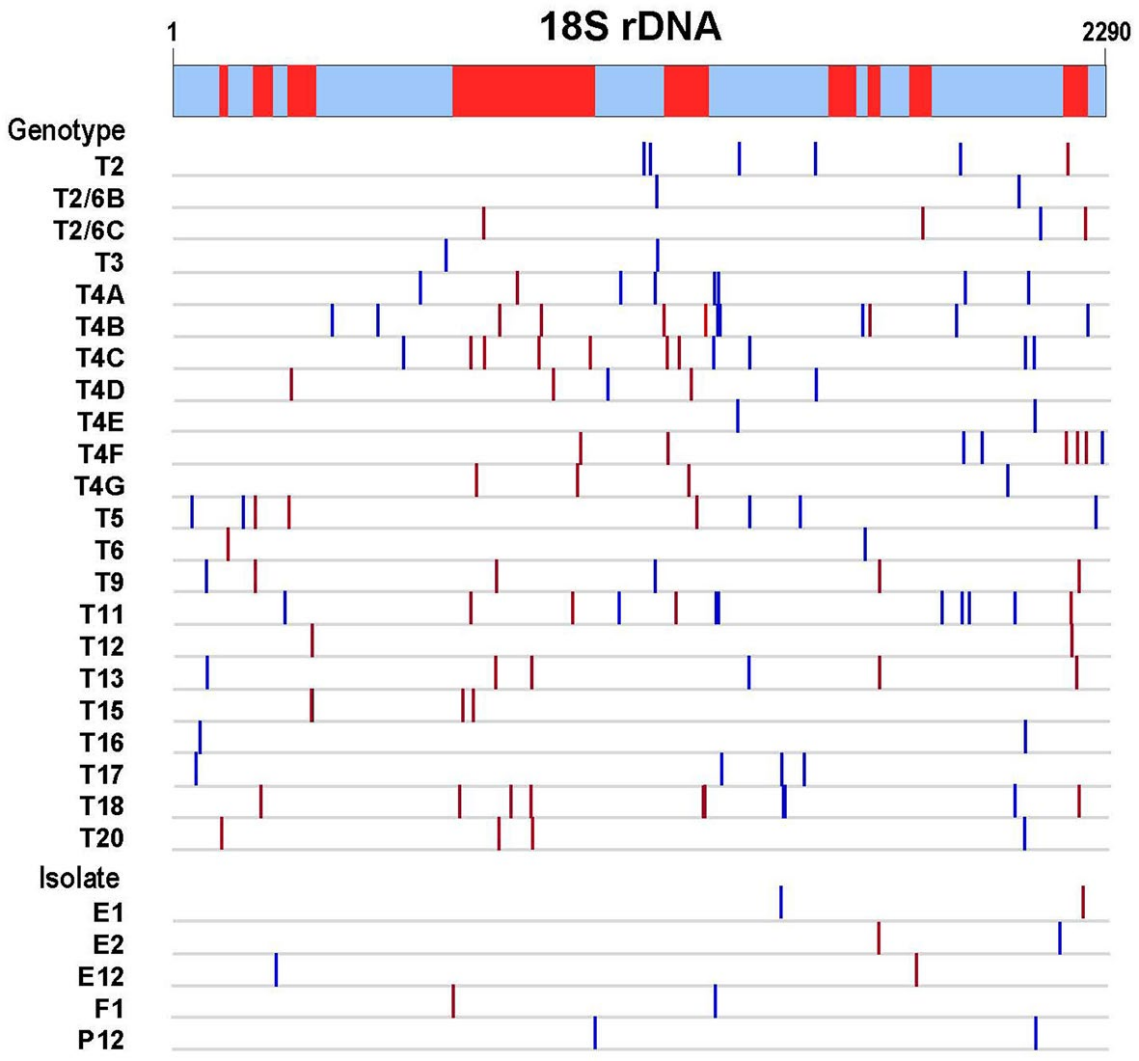
Recombination in the 18S rRNA sequences within genotypes and subtypes of *Acanthamoeba*. Vertical lines indicate the potential recombination breakpoints corresponding to sequence of *A. castellanii* (GenBank accession no. U07413). Unfilled and filled boxes in the scheme represent conserved and variable regions of the gene, respectively.

To investigate whether intragenic recombination contributed to sequence diversity in the extant 18S rRNA gene of *Acanthamoeba*, potential recombination breakpoints were analysed for each genotype and subtype of Thai isolates and previously reported sequences by using the Recombination Detection Program version 4 (RDP4)^30^. Genotypes T2/6C, T8, T14, T19 and T21 were excluded from analysis due to insufficient number of available sequences (n = 2 for each genotype with > 2 kb). Results reveal that potential recombination sites have been identified within genotypes/subtypes T2, T2/6B, T2/6C, T3, T4A-T4G, T5, T6, T9, T11-T13, T15-T18 and T20 (Fig. 3 and Supplemental Table S4). The potential recombination breakpoints were widely distributed across the 18S rRNA locus (Fig. 3). Meanwhile, analysis of 7 isolates bearing three or more variant sequences from our previous study has shown that 4 of these isolates belonging to genotypes/subtypes T4B, T4C, T9 and T17 contained recombination breakpoints in this locus^18^ (Fig. 3).

### Neutrality test

To determine whether the pattern of sequence diversity in the 18S rRNA gene of *Acanthamoeba* has been evolved neutrality or under natural selection, Tajima’s *D* and its related statistics, i.e. Fu & Li’s D* and F*, were deployed to analyse genotypes/subtypes T3, T4A-T4C, T4D, T4F and T5. Results revealed that almost all genotypes/subtypes yielded significant negative Tajima’s *D* values except subtype T4D. However, the Fu and Li’s D* and F* tests gave significant negative deviation from selective neutrality for all genotypes and subtypes (Table 4).

**Table 4:** Neutrality test for the 18S rRNA gene within genotypes and subtypes of *Acanthamoeba.*

Genotype/Subtype	N	No. sites§	M	S	H	*h* ± S.D	Tajima’s *D*	Fu & Li’s D*	Fu & Li’s F*
T3	25	1379	43	43	12	0.803 ± 0.078	− 1.8374 *	− 3.1296 **	− 3.1966 **
T4A	78	1329	159	149	55	0.982 ± 0.007	− 2.0658 *	− 6.5475 **	− 5.6421 **
T4B	91	2041	200	173	54	0.965 ± 0.010	− 1.9946 *	− 5.9575 **	− 5.1222 **
T4C	53	2055	176	166	42	0.984 ± 0.010	− 2.2137 ***	− 5.5094 **	− 5.0879 **
T4D	28	2065	76	76	19	0.881 ± 0.060	− 1.4964	− 3.0084 *	− 2.9664 *
T4F	40	2099	143	140	33	0.972 ± 0.019	− 2.2476 ***	− 5.1082 **	− 4.8659 **
T4 all	313	1321	414	349	201	0.992 ± 0.001	− 2.2828 ***	− 12.2621 **	− 8.2631 **
T5	31	2042	180	168	19	0.935 ± 0.030	− 1.9973 *	− 2.5526 *	− 2.8017 *

N, total number of taxons; M, total number of mutations; S, number of segregating sites; H, number of haplotypes; *h*, haplotype diversity.

^§^ excluding sites with gaps or missing data.

*P < 0.05; **P < 0.02, ***P < 0.01.

### The novel genotype ‘T23’

**Figure 4:**
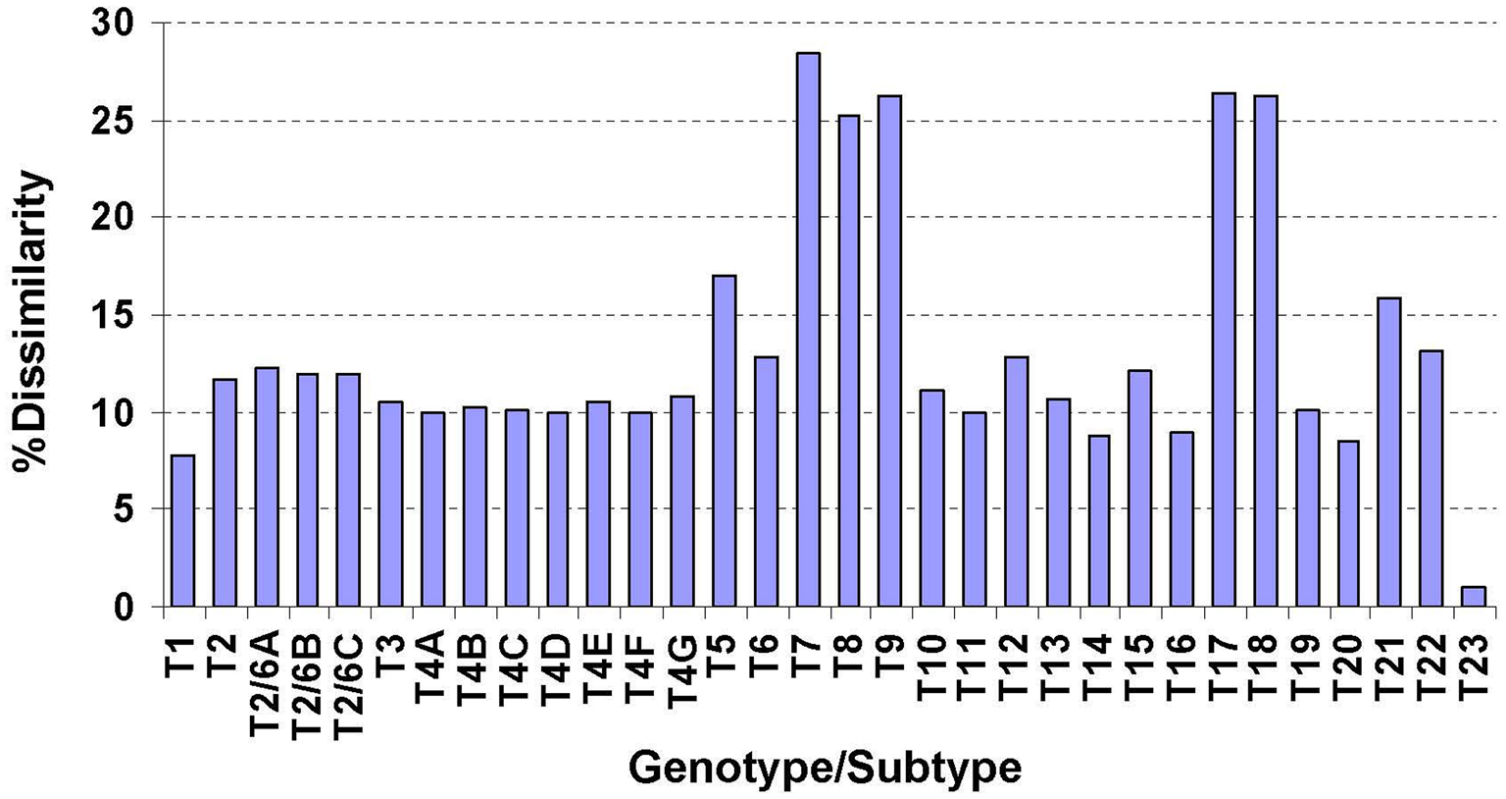
Percentages of nucleotide differences in the 18S rRNA gene of *Acanthamoeba* based on pairwise comparison between genotype T23 (AcW61A) and each representative known genotype. Dissimilarity within genotype T23 was determined from AcW61A and AcW61B sequences.

**Figure 5:**
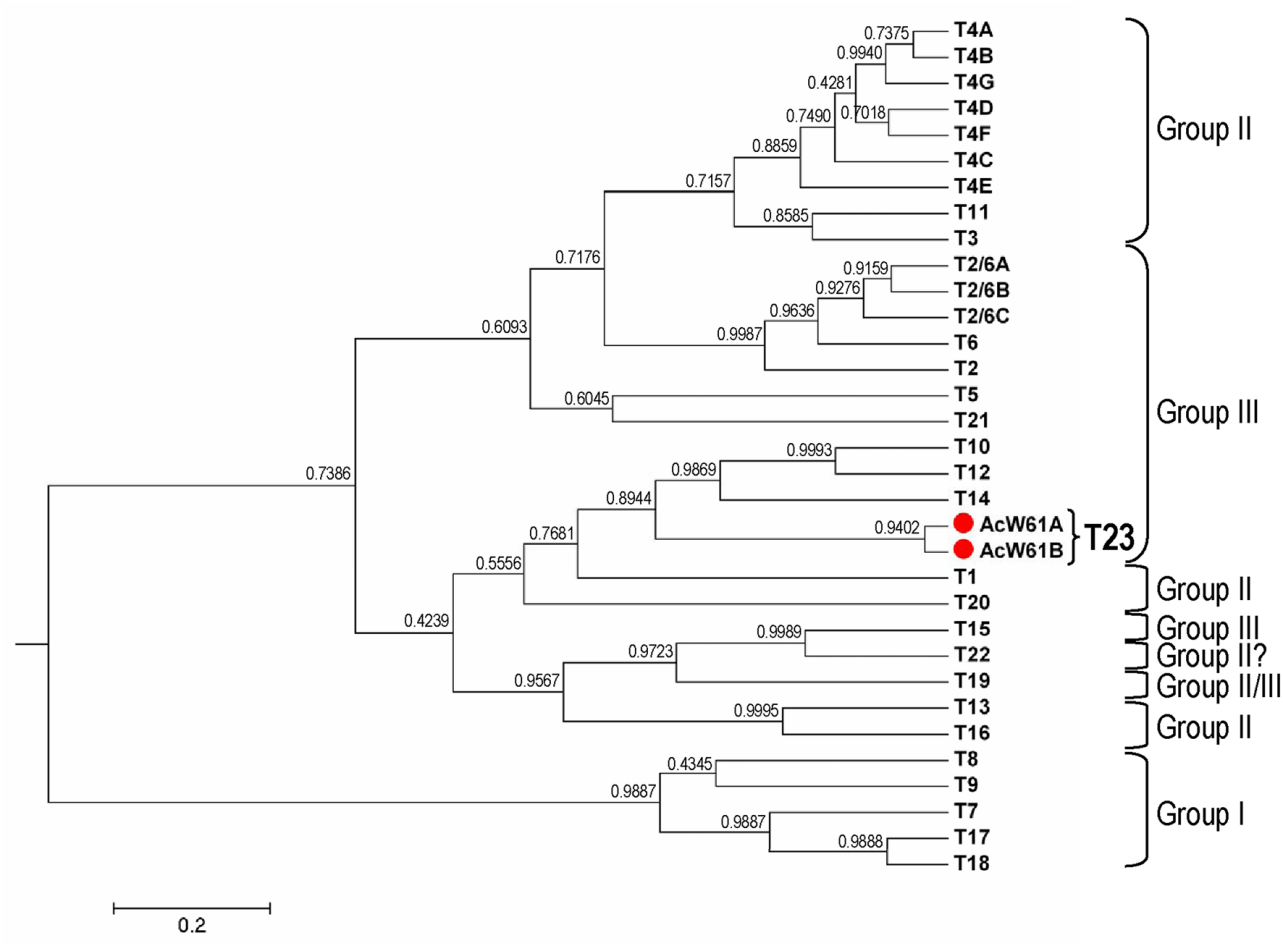
Bayesian phylogenetic tree inferred from the 18S rRNA sequences spanning > 2 kb of the novel genotype T23 (AcW61A and AcW61B, red spots) relative to all known genotypes of *Acanthamoeba*. The tree was visualized using the FigTree version 1.4.0 program^61^. Known morphological groups are shown corresponding to genotypes.

**Figure 6:**
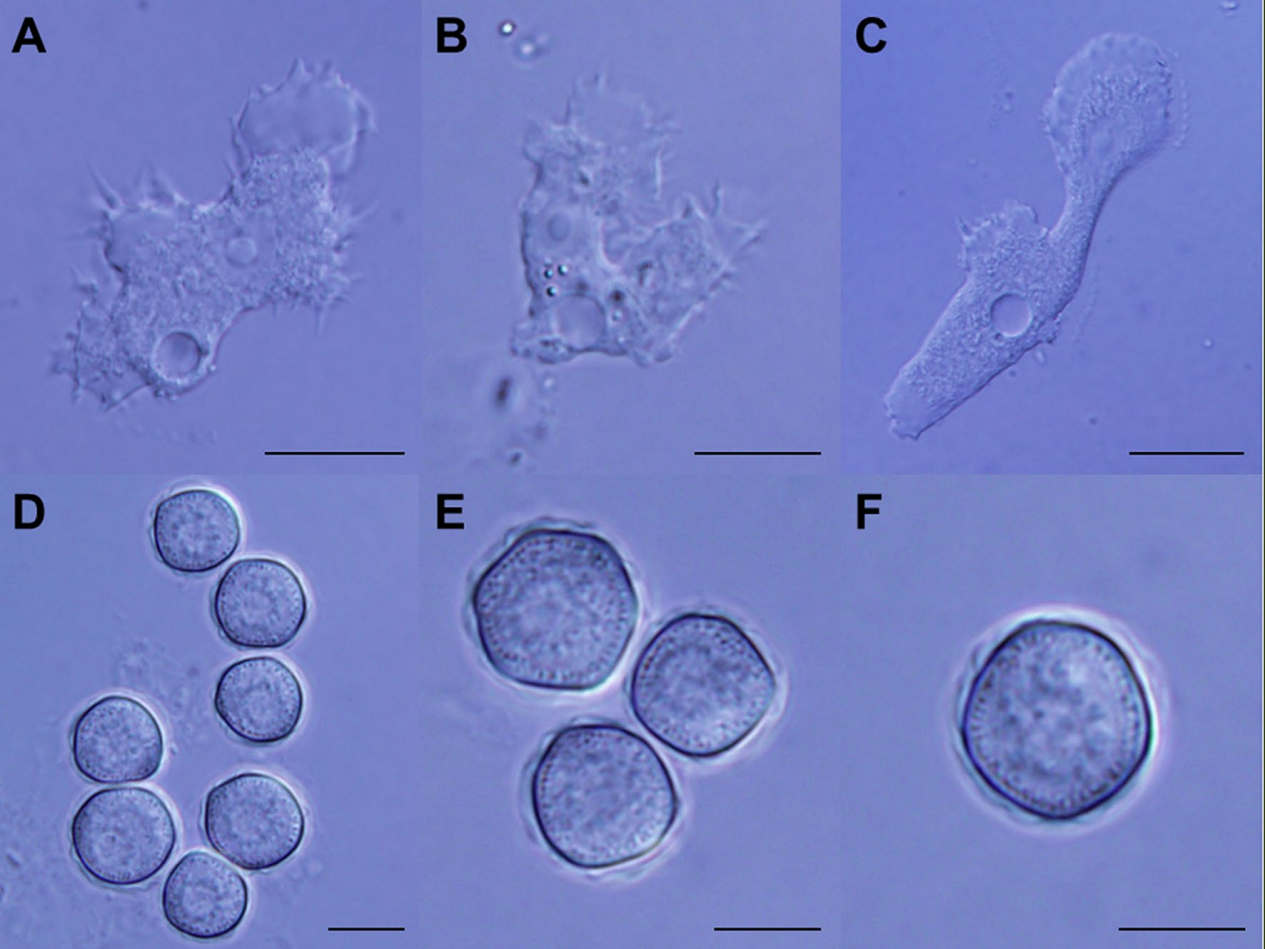
Photomicrograph of *Acanthamoeba* genotype 23 showing trophozoites of isolate AcW61 (**A** and **B**), a trophozoite in movement (**C**) and their cysts (**D**−**F**). Bar represents 10 μm.

Isolate AcW61 sampling from a pond inside Chatuchak District in Bangkok had unreadable electropherogram from nucleotide position 95 of the primary PCR amplicon onward upon initial direct sequencing of the PCR product. After limiting dilutions of the primary PCR templates, 2 distinct sequences (AcW61A and AcW61B) were obtained from direct sequencing of the secondary PCR products with clear electropherogram signals throughout the amplicons. A nucleotide deletion at position 96 of the AcW61B sequence relative to the AcW61A sequence seems to be responsible for the unreadable sequences of the primary PCR products. Re-cloning by single cyst isolation of isolate AcW61 followed by cultivation yielded the same results, suggesting allelic variation in the 18S rRNA gene copies of this isolate. Likewise, results from sequencing of recombinant plasmid clones from the primary PCR products of this isolate have reaffirmed AcW61A and AcW61B sequences. Importantly, the 18S rRNA sequences of AcW61A displayed 7.8% to 28.4% sequence dissimilarity in comparison with other known genotypes while AcW61A and AcW61B sequences shared 99.0% identity (Fig. 4 and Supplemental Table S4). Meanwhile, both Bayesian and maximum likelihood phylogenetic trees gave consistent results in which both AcW61A and AcW61B sequences seem to have diverged from genotype T14 while the percentages of sequence similarity/identity were highest when compared with genotype T1 (92.2%), followed by genotypes T20 (91.5%) and T14 ((91.3%), respectively (Figs. 4, 5 and Supplemental Fig. S3). The trophozoites of genotype T23 grows well on 1.5% non-nutrient agar seeded with heat-inactivated *E. coli* at ambient temperature ranging from 25 °C to 30 °C as well as at 37 °C while no growth is observed at 40 °C and 42 °C. The trophozoites are variable in shape and measure approximately 20–40 μm, with typical hyaline acanthopodia during movement. The endoplasm contains finely granular materials including bacteria upon feeding and one or a few large vacuoles. Each trophozoite possesses a single vesicular nucleus with a prominent centrally placed nucleolus while multiple (usually 2) nuclei can be observed during cellular division. Based on measurement of 100 cysts, the widest diameters range from 10 to 17 μm and the shortest from 9 to 16 μm with mean (± standard deviation) diameters of 12.95 ± 1.77 and 13.26 ± 1.82 μm, respectively (Fig. 6). The ectocyst is rather thin with seemingly smooth or slightly wrinkled surface. The endocyst displays relatively round shape while rounded corner pentagon and hexagon shapes are also observed. It is noteworthy that the cysts of isolate AcW61 belongs to morphological group III akin to those found in genotypes T10, T12 and T14. Taken together, the isolate AcW61 is considered to be a novel genotype of *Acanthamoeba*, designated ‘T23’.

## Discussion

Searching for *Acanthamoeba* in 100 public freshwater sources across 28 provinces of Thailand has identified 9 genotypes, comprising genotypes T2/6, T3-T5, T9, T11, T12, T18 and a novel genotype T23. Consistent with previous reports, genotype T4 predominated in this study, suggesting that *Acanthamoeba* carrying this genotype could be well adapted for survival in diverse ecological niche^4,15,31^. Despite the fact that different genotypes of *Acanthamoeba* exhibited variation in pathogenic traits, e.g. presence of serine protease, osmotolerance, thermotolerance and cytopathic effects, the ubiquitous existence of the T4 genotype in environment could partly be responsible for the high prevalence of this genotype as an important causative agent of human infections^31^. Further analysis has shown that the most predominant subtype within T4 in this survey belonged to T4B, followed by T4F, T4C and T4A whilst T4D, T4E and T4G were not detected. Although subtype T4A was observed in 4.6% of genotype T4, it is the most predominant subtype of worldwide isolates analysed so far, suggesting differential distribution of T4 subtypes in diverse environment^4,15,31^. It is noteworthy that genotype T10 occurring in a keratitis patient in Thailand could not be isolated from all water sources in this survey, suggesting that freshwater might not be a suitable habitat for this genotype or it could be rare in nature. The occurrence of identical near full-length 18S rRNA sequences of *Acanthamoeba* across regions of Thailand and from different period of sampling as well as the presence of identical sequences of isolates from Thailand with those from distant nations could suggest that some strains may successfully grow and occupy diverse ecological niche, albeit more comprehensive genetic analysis is required to address this issue (Supplemental Table S3). Meanwhile, intragenotypic nucleotide diversity of this locus among isolates in this study was significantly less than that among isolates elsewhere outside Thailand for genotypes T3, T9, T11 and subtypes T4A-T4C, T4F and T2/6B, implying that the magnitude of sequence diversity within local populations seems to be relatively limited (Table 3).

To date, a subset of the 18S rRNA sequences of genotypes T3, T4A, T 5 and T15 contains group I intronic insertions at specific locations in conserved domains^13,25–27,32^. Among genotypes T3, T4A and T5 identified in this study, only genotype T3 contained intronic insertion corresponding to S516. Together with sequences available in GenBank database, 2 of 5 intronic variants in genotype T3 have been newly identified in this study (Supplemental Figs. S1 and S2). It is noteworthy that insertion or deletion of nucleotides mainly contributed to sequence variation in intronic sequences and some of which were located in the regions containing homo- or dimeric short tandem repeats (Supplemental Fig. S1). It is likely that slippage strand mispairing mechanism could generate sequence variation in the intron of genotype T3. Meanwhile, the origin of introns has been suggested to be ancient and could have co-evolved with rRNAs. The presence of introns in the same locations of the rRNA genes across domains of life could imply that their existence predated evolutionary divergence and speciation^33^. It has been suggested that the wide spread occurrence of the S516 intron in various eukaryotes could have arisen from vertical transfer while subsequent loss of the intron could occur later in the evolution^29,33^. Although intron gain and loss could occur in rRNAs, the lack of intron in all isolates bearing genotypes T4 and T5 in this study could suggest that the former process may not be common among *Acanthamoeba* bearing these genotypes in Thailand.

Analysis of the 18S rRNA gene of isolate AcW61 has revealed 2 related sequences, i.e. AcW61A and AcW61B, that could not be assigned to any known genotype. Both sequences shared 99.0% sequence identity, indicating that they belonged to the same genotype. The 18S rRNA sequences of isolate AcW61 exhibited more than 7.8% sequence differences from 22 known genotypes. The Bayesian phylogenetic tree has shown that the AcW61A and AcW61B sequences were located in a separate branch with posterior probability support approaching 0.9 from genotype T14 while both sequences were more related with genotypes T10, T12 and T14 than other genotypes. Furthermore, the cyst structure of isolate AcW61 shared the same morphological group III as those in the clade for genotypes T10, T12 and T14, suggesting that these genotypes could have arisen from the common ancestor. Although the thermotolerance property of *Acanthamoeba* seems not to be directly related with its pathogenicity, several clinical isolates were capable of growing at 37 °C or at higher temperature^23,34–36^. Although the novel genotype T23 grew well at 37 °C, it remains to be explored whether this genotype is pathogenic to humans.

One of the complex characteristics of protozoans in the genus *Acanthamoeba* is the presence of polyploid genome that contain as many as 25n. Each haploid genome of *Acanthamoeba* contains 24 copies of the 18S rRNA gene or ~ 600 gene copies per cell^37^. The 18S rRNA gene of *Acanthamoeba* spanning > 2 kb in this study exhibited extensive sequence diversity among genotypes that stemmed from both nucleotide substitutions and insertions/deletions. Despite extensive variation in the available number of sequences in each genotype analysed (Table 3), it seems that the levels of nucleotide diversity within genotypes were > 0.01 except genotype 17 which could be due to the low number of available sequences (n = 6). Interestingly, genotype T11 displayed 1.7–4.8 times higher level of nucleotide diversity than other geneotypes, suggesting the existence of subtypes among isolates bearing this genotype. Accordingly, maximum likelihood tree has shown 2 distinct clades within genotype T11 akin to those found as subtypes in genotype T4, implying that genetic diversity and evolutionary divergence within genotypes of *Acanthamoeba* could be more than currently perceived if more isolates are analysed (Supplemental Fig. S4).

The organization of the multicopy rRNA gene clusters of most prokaryotes and eukaryotes are found in tandem arrays with head-to-tail arrangement. Several lines of evidence have suggested that each gene copy has been generated by the process of concerted evolution in which unequal crossover occurs randomly among gene members^38,39^. According to the model, repeated events of unequal crossover or gene conversion have homogenized the gene members. Therefore, identical or near identical sequences among the rRNA gene members can be found when erroneous incorporation of nucleotide during nuclear division is absence or low. The clear non-superimposed signals in the electropherograms of most *Acanthamoeba* isolates in this study have suggested that the majority of gene members in the rRNA gene shared identical sequences. Mixed sequences observed in 6 isolates in this study could be from coexistence of subpopulations bearing distinct haplotypes or intragenomic heterogeneity of the 18S rRNA sequences. However, the extent of sequence dissimilarity between distinct haplotypes within isolates upon pairwise comparison was consistently minimal (less than 2%), suggesting intragenomic allelic heterogeneity of this locus. Importantly, the presence of multiple 18S rRNA sequences within cloned *Acanthamoeba* samples has been previously observed by Stothard and colleagues who found 7 of 53 *Acanthamoeba* strains harboured different alleles within cloned samples, implying that individual *Acanthamoeba* cell could have multiple 18S rRNA haplotypes albeit belonging to the same genotype^12^. In this study, the 18S rRNA sequences of most isolates (125 of 131) could be determined by direct sequencing of the PCR product, suggesting that the majority of gene members in this locus of most isolates harboured identical sequences or the presence of predominant haplotypes among members in this gene family precluding detection of minor variants upon direct sequencing of the amplicons. Heterogeneity of sequences among 18S rRNA gene members could have arisen during the process of DNA replication by point mutations or slippage strand mispairing between repetitive sequences leading to insertion or deletion of some nucleotides.

Meanwhile, analysis of the available > 2 kb 18S rRNA sequences of genotypes T2, T3-T5, T6, T9, T11-T13, T15-T18 and T20, and subtypes T2/6B and T2/6C has revealed that intragenic recombination occurred within these genotypes and subtypes. Furthermore, recombination breakpoints have been identified across the 18S rRNA gene, suggesting recombination has occurred at random. It is noteworthy that *Acanthamoeba* reproduces asexually by binary fission although highly expressed meiotic genes have been discovered in this organism, leading to a suspicion of cryptic sexual lineages^40,41^. Recombination in the 18S rRNA gene of *Acanthamoeba* could be generated between gene members carrying distinct sequences; thereby, recombinant sequences were observed in some sequences within genotypes and subtypes. It is noteworthy that the total rate of spontaneous intrachromosomal unequal crossovers in *Saccharomyces cerevisiae* has been estimated to be 1% per mitotic division which was more frequent than interchromosomal exchanges during meiosis^42–44^. Therefore, the lack of sexual stage in *Acanthamoeba* seems not to preclude genetic recombination in the 18S rRNA locus. The presence of recombination breakpoints in individual gene members within isolates bearing genotypes T4B, T4C, T9 and T17 from our previous study^18^ was in accord with intragenomic recombination in the rRNA locus as reported in other organisms^45–48^. The high copy number of the 18S rRNA gene per genome, as in *Acanthamoeba*, could promote intrachromosomal crossovers while emerging mutations, albeit at a slow rate in each unit, could spread across all gene members or be eliminated by unequal crossovers or gene conversion^37,49^. Furthermore, the number of repeat units in the rRNA locus could also be increased or decreased after multiple rounds of recombination events^49,50^.

Intragenomic sequence variation among individual member of the rRNA gene has been discovered in various organisms, implying that natural selection may also operate on this gene family^45,51–53^. Sequence variation in the 18S rRNA locus seems to have evolved under different directions of natural selection, probably depending on the functional requirement of organisms or their life histories. For example, intragenomic rRNA polymorphisms in nematodes have been suggested to evolve neutrally^52^ whereas sequence variation in the rRNAs associated with ‘stress-response ribosome’ in *Escherichia coli* seems to be in line with positive selection^54^. Accordingly, rRNA variants conferring fitness would be maintained by positive selection whilst genetic drift producing deleterious effects could be eliminated by purifying selection^39,51^. Meanwhile, Tajima’s *D* and its related statistics, Fu and Li’s D* and F* tests have detected significant negative direction of deviation from selective neutrality in the 18S rRNA gene of *Acanthamoeba* (Table 4), implying that negative or purifying selection has influenced sequence diversity at this locus. The uniformity of significant negative values of Tajima’s *D* and/or its related statistics across genotypes and subtypes has further suggested that purifying selection could predate genotypic diversification of *Acanthamoeba*. The role of purifying selection could be an important mechanism to preserve a specific nucleotide sequence as well as its functional property of the rRNA transcripts while deleterious mutations would be subject to elimination.

In conclusion, surveys of freshwater sources across diverse regions of Thailand along with our previous report have revealed at least 11 genotypes of *Acanthamoeba* in this country^18^. Of these, a novel genotype, designated ‘T23’, bearing cyst structure conforming to morphological group III has been identified. Phylogenetic tree has placed the ‘T23’ genotype within the cluster of genotypes T10, T12 and T14, all of which possess morphological group III cysts. Among intron-containing genotypes, the majority of Thai isolates (15 out of 17) belonging to genotype T3 possessed 4 variants of group I intron located at S516. The presence of identical near complete 18S rRNA sequences across regions of Thailand and across nations has suggested that some strains have reproductive success to occupy diverse ecological niche. The extent of nucleotide diversity of genotype T11 exceeded those of other genotypes, implying the presence of subtypes within this genotype. Evidence for intragenic recombination in the 18S rRNA gene has been detected across genotypes of *Acanthamoeba* whilst purifying selection seems to operate on this locus. It is likely that more novel genotypes of *Acanthamoeba* remain to be discovered either from environmental sources or clinical specimens.

## Taxonomic summary

Phylum Amoebozoa (Lühe, 1913 emend. Cavalier-Smith, 1998).

Subphylum Lobosa (Carpenter, 1861 emend. Cavalier-Smith, 2009).

Class Discosea (Cavalier-Smith, 2004).

Subclass Longamoeba (Smirnov & Cavalier-Smith, 2011).

Order Centramoebida (Rogerson & Paterson, 2002 emend. Cavelier-Smith, 2004).

Family Acanthmoebidae (Sawyer & Griffin, 1975).

Genus *Acanthamoeba* (Volkonsky, 1931).

Genotype: T23.

Species: *Acanthamoeba bangkokensis* sp. nov.

Diagnosis. The near complete 18S rRNA sequences of isolate AcW61 contains 2 highly related sequences and are sister to genotype T14 on a phylogenetic tree. The trophozoites are not distinguishable from other genotypes while the cyst structure conforms with Pussard and Pond’s morphological group III with an average diameters 13.0 by 12.7 μm (range 10–17 × 9–16 μm). The phylogenetic tree inferred from the ASA.S1 fragment of the 18S rRNA gene can differentiate and assign this genotype similar to the tree constructed by using the near complete sequence of this locus. Isolate AcW61 can grow well at ambient temperature as well as at 37 °C on 1.5% non-nutrient agar overlaid with *E. coli*.

### Etymology

*Acanthamoeba*, a genus name of pathogenic free-living amoeba with cosmopolitan distribution; *bangkokensis*, originating in Bangkok, the capital city of Thailand where this species was first isolated from a public freshwater pond in Chatuchak District.

### Type species

*Acanthamoeba bangkokensis* sp. nov., strain AcW61, morphological group III, genotype T23.

### Gene sequence data

The 18S rRNA sequence has been deposited in GenBank database under accession numbers MZ272148 and MZ272149.

## Materials and methods

### Samples

Water samples were collected from 100 public water sources including natural or artificial ponds and lakes (n = 72), rivers (n = 5), water falls (n = 11), irrigation canals (n = 7), moats (n = 3) and fountains (n = 2) in 28 provinces of Thailand (Fig. 1A and Supplemental Table S1). These water sources were selected because they were near residential areas or in public places where people could easily gain access to them. Water samples were taken approximately 20 to 50 cm from the edge of the water sources and 10 to 20 cm in depth using 500 ml sterile polypropylene bottles. The number of samples collected depending on the approximate surface area of each site, i.e. 5 and 10 samples for those with surface area < 500 and > 500 square meters, respectively. For each site, the water samples were collected covering a distance of approximately 5 m interval. The survey period was from January o March 2015. Temperature and pH of water surface were recorded at the time of sampling.

### Cultivation of *Acanthamoeba*

Each freshwater sample was centrifuged at 750 × *g* for 10 min. The supernatant was discarded and the remaining pellet was applied onto 1.5% non-nutrient agar overlaid with 2 ml of milky suspension of heat inactivated (60 °C, 30 min) *Escherichia coli*. The culture was incubated at room temperature, varying from 25 °C to 30 °C, and examined daily for 7 consecutive days under an inverted microscope (Olympus IX71, Tokyo, Japan). Positive cultures for trophozoites of *Acanthamoeba* were incubated further until the characteristic cysts appeared. *Acanthamoeba* was isolated by cutting off small pieces of agar surface containing the characteristic cysts and transferred them into 1 ml of 1% sulphuric acid and kept over night at ambient temperature to remove other free-living organisms in water. After centrifugation at 750 × g for 10 min, the sediment was used for additional rounds of the same culture condition until *Acanthamoeba* became overpopulated in the culture. Each *Acanthamoeba* isolate was obtained from single cyst isolation and subject to the same culture condition. For thermotolerance testing of some isolates, the cultures were incubated at ambient temperature, 37 °C, 40 °C and 42 °C.

### Amplification of the 18S rRNA gene

Trophozoites of *Acanthamoebae* were harvested from in vitro culture originated from established isolates and centrifuged at 750 × *g* for 10 min. DNA was extracted from the remaining pellet using QIAamp DNA mini kit (Qiagen, Hilden, Germany). The nuclear small subunit rRNA gene encompassing > 2 kb was amplified by PCR using primers ACAN18SF0: 5’-TCCTGCCAGTAGTCATATGC-3’ (nucleotides 9–28 of *A. castellanii* Neff strain, GenBank accession number U07416) and ACAN18SR0: 5’-CTTCTCCTTCCTCTAAATGGT-3’ (nucleotides 2236–2256) as described previously^18^. Although the primers are not specific for *Acanthamoeba 18S rRNA*, the abundant genomic DNA templates from the pre-cultured *Acanthamoeba* isolates have allowed efficient amplification of the target gene. DNA amplification was carried out in a total volume of 30 µl of the reaction mixture containing template DNA, 2.5 mM MgCl_2_, 300 mM each deoxynucleoside triphosphate, 3 µl of 10X ExTaq PCR buffer, 0.3 µM of each primer and 1.25 units of DNA polymerase. Thermal cycling profiles included the preamplification denaturation at 94 °C for 1 min followed by 35 cycles of 94 °C for 40 s, 55 °C for 30 s and 72 °C for 2 min, and a final extension at 72 °C for 5 min^18^. DNA amplification was performed by using a GeneAmp 9700 PCR thermal cycler (Applied Biosystems, Foster City, CA). To minimize sequence error during PCR amplification, ExTaq DNA polymerase was deployed which possesses efficient 3’-to-5’ exonuclease activity for fidelity without strand displacement (Takara, Shiga, Japan).

### Direct sequencing

The PCR products were purified by using QIAquick PCR purification kit (Qiagen, Hilden, Germany) and used as templates for sequencing. DNA sequences were analysed from each template using the Big Dye Terminator v3.1 Cycle Sequencing Kit on an ABI3100 Genetic Analyser (Applied Biosystems, USA). Sequencing primers were used to obtain overlapping sequences^18^.

### Limiting dilution of PCR template and sequencing

For isolates containing superimposed signals on electropherogram from direct sequencing of the purified PCR products which could suggest either mixed genotypes or the presence of heterogeneous 18S rRNA templates within each isolate^12,18^. The PCR-amplified 18S rRNA gene products of these isolates were subject to tenfold serial dilutions and used as DNA templates for secondary PCR. The secondary PCR was performed essentially the same as the primary PCR except that the semi-nested PCR primers ACAN18SF1: 5’-GCTTGTCTCAAAGATTAAGC-3’ (nucleotides 24–46) and ACAN18SR0 were used and the amplification was done for 25 cycles. Secondary PCR products generated from the highest dilution of primary PCR products were used as templates for direct sequencing. These serial dilutions, secondary PCR and sequencing were performed in 5 separate batches for each isolates whose sequence contained superimposed signal on electropherogram.

### Subcloning and sequencing

The PCR products of isolate AcW61 were excised from agarose gel, purified by using a QIAquick PCR purification kit (Qiagen), and ligated into pGEM-T-Easy Vector (Promega, Madison WI, USA) as described previously^18,55^. After incubation at 4 °C for overnight, the reaction mixture was precipitated, dissolved in 10 µl of double-distilled water, and transformed into *Escherichia coli* strain JM109. DNA sequences were determined from at least 5 recombinant subclones of each isolate.

### Data analysis

For genotype assignment, the sequences were aligned according to their secondary structure by using the R-COFFEE program^56,57^. The 18S rRNA sequences of representative genotypes, species, strain and GenBank accession numbers of *Acanthamoeba* used as references included T1, *A. castellanii* V006 ATCC 50494 (U07400), T2, *A. palestinensis* Reich ATCC 30870 (U07411); T2/6A, *A. polyphaga* CCAP 1501/3b ATCC 30872 (AY026244); T2/6B, *A.* sp. OB3b_3A (AB425945); T2/6C, *A. polyphaga* OX-1 CCAP 1501/3c (AF019051); T3, *A. griffini* S-7 ATCC 30,731 (U07412); T4A, *A. castellanii* ATCC 30011 (U07413); T4B, *A. castellanii* Ma ATCC 50370 (U07414); T4C, *A.* sp. Fernandez ATCC 50369 (U07409); T4D, *A. rhysodes* Singh ATCC 30973 (AY351644); T4E, *A. polyphaga* Page-23 ATCC 30871 (AF019061); T4F, *A. triangularis* SH621 ATCC 50254 (AF346662); T4G, *A. castellanii* Neff ATCC 30010 (U07416); T5, *A. lenticulata* Jc-1 (U94739); T6, *A. palestinensis* 2802 ATCC 50708 (AF019063); T7, *A. astronyxis* Ray & Hayes ATCC 30137 (AF019064); T8, *A. tubiashi* OC-15C ATCC 30867 (AF019065); T9, *A. comandoni* ATCC 30135 (AF019066); T10, *A. culbertsoni* Lilly A-1 ATCC 30171 (AF019067); T11, *A. hatchetti* BH-2 ATCC 30730 (AF019068); T12, *A. healyi* ATCC 30866 (AF019070); T13, *A.* sp. UWC-9 ATCC PRA-3 (AF132134); T14, *A.* sp. PN13 (AF333609); T15, *A. jacobsi* AcaP13_cl2 (KY513791); T16, *A.* sp. U/H-C1 (AY026245); T17, *A.* sp. Ac-E1a (GU808277); T18, *A. tubiashi* CDC,V621 (KC822461); T19, *A.* sp. USP-AWW-A68 (KJ413084); T20, *A.* sp. OSU 04–020 (DQ451161); T21, *A. pyriformis* CR15 (KX840327) and T22, *A. royreba* ATCC30884 (CDEZ01000000). Phylogenetic trees were constructed using maximum-likelihood method and Bayesian approach. Based on analysis of the best model for the sequence data, the maximum likelihood tree was constructed using Tamura-Nei model of nucleotide substitution in which gamma model was used for correction of multiple hits by taking into account the variation of nucleotide frequencies and differences in substitution rate between nucleotides^58^. Reliability of the tree branching patterns was analysed by 1,000 bootstrap pseudoreplicates using the MEGA 6.0 program^59^. Bayesian phylogenetic analysis was performed based on Markov Chain Monte Carlo algorithm with uncorrelated lognormal relaxed clock, coalescent constant population and a 4 category gamma site heterogeneity model. Simulations were run for 20,000,000 cycles and logged at every 1,000 cycles using the Bayesian Evolutionary Analysis by Sampling Trees 2 (BEAST 2) package^60^. Maximum clade credibility tree and mean heights were used to determine posterior probability of trees implemented in the TreeAnnotator program. Tree topology was visualized using the FigTree version 1.4.0 program in the BEAST 2 package^61^. For analysis of haplotype and nucleotide diversity, sequences of each genotype or subtype were aligned separately by using the MAFFT version 7 with manual adjustment^62^. Nucleotide diversity was computed by using the Tamura-Nei substitution model and gamma distribution of evolutionary rate differences among sites whilst the standard error was determined from 1000 bootstrap pseudoreplicates using the MEGA version 6.0 program^59^. Differences between nucleotide diversity values were tested by a two-tailed Z-test and the significance level was set at *P* < 0.05. Percentage of similarity/identity between sequences was calculated by using the Sequence Manipulation Suite^63^. Analysis of intragenic recombination was performed by using the Recombination Detection Program version 4 (RDP4)^64^ that included 3SEQ^65^, Bootscan/Recscan^30^, CHIMAERA^66^, GENCONV^67^, the Maximum Chi Square^68^, RDP4^30^ and Sister Scanning^69^ methods. To compare the extent of sequence variation within genotypes between Thai and global isolates, 350 sequences containing > 2 kb deposited in the GenBank database were retrieved for comparison. For genotypes with few sequences available, sequences > 1.4 kb were also included for analysis (Supplemental Table S2).

### Accession numbers

One hundred and thirty seven near complete sequences of the 18S rRNA gene of *Acanthamoeba* have been deposited in NCBI GenBank under accession numbers MZ272072-MZ272208.

## Supplementary Information

Supplementary Information.

## Data Availability

The datasets generated during and/or analyses during the current study are available from the corresponding author upon request.
