# Supplementary material for: Analysis of Acanthamoeba genotypes from public freshwater sources in Thailand reveals a new genotype, T23 Acanthamoeba bangkokensis sp. nov
Source: Sci Rep. 2021 Aug 27;11:17290. doi: 10.1038/s41598-021-96690-0 (PMC8397737; doi:10.1038/s41598-021-96690-0)

Analysis of *Acanthamoeba* genotypes from public freshwater sources in Thailand reveals a new genotype, T23 *Acanthamoeba bangkokensis* sp. nov.

Chaturong Putaporntip, Napaporn Kuamsab, Warisa Nuprasert, Rattanaporn Rojrung, Urassaya Pattanawong, Taweesak Tia, Surasuk Yanmanee, Somchai Jongwutiwes*

Molecular Biology of Malaria and Opportunistic Parasites Research Unit, Department of Parasitology, Faculty of Medicine, Chulalongkorn University, Bangkok, Thailand.

SUPPLEMENTARY INFORMATION

Contents

Table S1. Information on water sampling sites and *Acanthamoeba* isolates…..………..…..….2

Table S2. Isolates with identical 18S rRNA gene sequences across > 2 kb and their geographic origins……………………………………………………….……….…....6

Table S3. Recombination in the 18S rRNA sequences within genotypes/subtypes and isolates of *Acanthamoeba*……………………………………………………..……….....7

Table S4. Percentages of sequence identity between AcW61A and representative genotypes including AcW61B…………………………………………….…………….....11

Table S5. *Acanthamoeba* 18S rRNA sequences from public database and their geographic origins..……………………………………………………………………….........12

Figure S1. Variation in intron of genotype T3………………..……………………..…………24

Figure S2. Secondary structure of intron in genotype T3…………………………..…..……25

Figure S3. Maximum likelihood tree inferred from the 18S rRNA sequences of representative genotypes and subtypes of *Acanthamoeba*………………..…………26

Figure S4. Maximum likelihood tree inferred from sequences of genotype T11, representative genotype T3 and subtypes T4A-T4G………………………..……27

Supplemental Table S1. Information on water sampling sites and *Acanthamoeba* isolates.

Region	Province	Place	GPS	pH	Temp (°C)	Isolate
North	Chiang Mai	Angkaew, Chiang Mai University	E98° 57' 00" N18° 48' 19"	7.7	29	AcW91, AcWT57
		Hueykaew Waterfall	E98° 56' 40" N18°48' 42"	6.8	26	
		Moat, Taepae	E98° 59' 36" N18° 47' 18"	8	30	AcWT93A, AcWT93B, AcW140
		Moat, Changpuek	E98° 59' 12" N18° 47' 43"	8.2	30	AcW92, AcW159
		Irrigation canal	E98° 57' 37" N18° 48' 00"	8	29	AcWT95, AcwT59
		Tungtao reservoir	E98° 56' 25" N18° 52' 05"	7.5	28	AcWT96
		Chaloem Phra Kiat 80 Phansa Park	E98° 57' 50" N18° 50' 03"	8	30	AcWT97
		Huay Kaew Reservoir	E98° 57′ 2″ N18° 50′ 1″	7.6	28	AcWT94
		Wat Sun Phasak Worra Au-Rai	E98° 56' 05" N18° 42' 52"	8	29	AcWT60, AcW144
	Lampang	Pond, Wat Phra That Lampang Luang	E99° 23' 19" N18° 13′ 03″	7.9	30	AcW139A, AcW139B
		Chaloem Phra Kiat 84 Phansa Park	E99°29'59.0" N18°17'52.1"	7.7	29	
		Nhong Krathing Park	E99° 28' 12" N18° 18' 13"	7.7	29	
	Tak	Rajamangala University of Technology Lanna - Tak	E99°07'20.6" N16°53'53.5"	7.6	30	AcWT47
		Ko loy Mani Banphot Swamp	E99°07'39.3" N16°52'31.6"	8	32.5	
		Mani Banphot Swamp	E99°07'42.3" N16°52'31.1"	7.8	31	AcW154
		Ping River	E99°07'23.7" N16°52'09.9"	7.9	29	
	Phitsanulok	Nan River	E100°15'36.1" N16°49'19.0"	7.7	32.5	
		Royal Monument of King Naresuan	E100°11'27.7" N16°44'57.2"	8.5	32	
		Pond, Faculty of Science, Naresuan University	E100°11'33.1" N16°44'33.4"	8	31	AcWT51
		Pond, Faculty of Public Health	E100°11'25.6" N16°44'41.5"	8.2	31	
		Chaloem Phra Kiat 80 Phansa Park	E100°17'22.6" N16°49'10.8"	7.8	30	AcW22, AcwT23
		Bueng Ratchanok Park	E100°21'20.1" N16°48'31.9"	7.8	30	AcW105
Central	Ayutthaya	Muang canal	E100°33'32.8" N14°21'38.8"	8.1	33	AcWT63, AcWT98, AcW162
		Ayothaya Floating Market	E100°35'36.5" N14°21'31.7"	8	32	AcWT99
	Chai Nat	Chainat Bird Park	E100°09'06.9" N15°12'24.9"	8	31	AcW146
		Chao Phraya River	E100°07'23.2" N15°11'01.6"	7.9	29	

Region	Province	Place	GPS	pH	Temp (°C)	Isolate
	Nakhon Sawan	Paradise Park	E100°07'20.1" N15°41'53.3"	8	30	AcW121, AcW122A, AcW122B, AcW157, AcWT53
		Swamp, Bueng Boraphet	E100°15'00.1" N15°41'00.7"	7.6	30	AcWT52, AcW145
	Ang Thong	Thusop Irrigation canal	E100°26'37.9" N14°35'17.3"	7.7	31	
		Somdej Pra Chao Tak Sin Maharat Public Park	E102°06'21.3" N12°36'20.8"	7.8	31	AcW147
	Bangkok	Benjakitti Park	E100°33'27.6" N13°43'48.6"	7.7	31	
		Benchasiri Park	E100°34'02.8" N13°43'50.6"	7.7	30	
		Saranrom Palace Park	E100°29'42.0" N13°44'53.4"	7.8	30	AcW132, AcW109A, AcW109B, AcW133
		The Train Rod Fai park	E100°33'15.1" N13°48'59.8"	7.6	30	AcWT48, AcWT6, AcWT64, AcWT100
		Chatuchak Park	E100°33'12.2" N13°48'27.9"	8.2	29	AcW61
		Thonburirom Park	E100°29'29.3" N13°39'07.9"	7.7	32	
		By the Pond, King Ramkhamhaeng Courtyard	E100°37'05.3" N13°45'27.8"	7.4	31	
		Suan Luang Rama IX	E100°39'40.2" N13°41'16.2"	8.1	29	AcWT1, AcWT58, AcWT65, AcW110A, AcW110B
		Nagaraphirom Park	E100°29'24.4" N 13°44'49.0"	7.8	30	AcW161
		Phasi Charoen canal	E100°21'39.5" N13°41'05.3"	7.9	30	
		Courtyard by the Lake Muang Thong Thani	E100°32'41.6" N13°55'15.5"	7.7	31	
		Thawi Wanarom Park	E100°21'09.3" N13°44'45.4"	7.5	30	AcWT32
		Lumphini Park	E100°32'29.1" N13°43'52.3"	7.6	30.5	AcWT66
East	Chanthaburi	Chanthaburi Provincial Rama 9 Commemoration Public Park	E102°05'09.4" N12°35'32.5"	7.7	32	AcW148
		Waterfall, Namtok Phlio National Park	E102°11'03.2" N12°31'46.9"	6.3	27	
		Trok Nong Water Fall	E102°14'13.6" N12°32'40.0"	6.4	26.5	AcWT40
		Khao Ra Kam Reservoir	E102°24'56.9" N12°15'27.6"	7.4	29	AcW149
	Trat	Sra Si Siat Reservoir	E102°29'50.6" N12°14'27.2"	7.5	29	AcWT50
		Trat Provincial Botanical Garden	E102°31'02.3" N12°15'19.1"	7.8	30	AcWT74
		Trat Provincial Botanical Garden	E102°31'03.6" N12°15'08.8"	7.9	31	AcWT49, AcW135
		Princess Mothers Garden	E101°04'00.9" N13°41'17.0"	8	31	AcW107, AcWT20
	Chachoengsao	pond, Chachoengsao Provincial	E101°04'10.8" N13°41'16.7"	7.5	30	AcWT55, AcW104

Region	Province	Place	GPS	pH	Temp (°C)	Isolate
		Administration City Hall				
		Water Supply and Maintenance Project Chaiyanuchit, Royal Irrigation Department	E101°04'39.0" N13°41'40.6"	7.5	30	AcW158
		Bang Pakong River	E 101°04'49.3" N 13°41'24.5"	7.6	29	
Northeast	Buri Ram	Wat Nong Bua Thong	E103°09'33.1" N 15°03'19.8"	7.6	31.5	AcW150
		La Lom canal	E103°06'37.8" N14°59'29.8"	7.5	31	
	Si Sa Ket	Park of the Duan Princess Mother Sisaket	E104°18'19.0" N15°06'10.0"	7.7	32	AcWT45, AcW160A, AcW160B
		Pond, Sisaket Rajabhat University	E104°21'34.6" N15°06'59.0"	7.7	31	
	Ubon Ratchathani	Thung Sri Mueang	E104°51'23.8" N15°13'46.5"	7.6	30	AcW134
		Fountain, Thung Sri Mueang	E104°51'26.0" N15°13'48.7"	8.3	33	
		Nong Bua Pond	E104°50'24.6" N15°15'42.6"	8.2	31	AcW156
	Roi Et	Pond, Sala Loy Park	E103°56'25.4" N16°01'49.1"	8	33	AcWT101, AcW118A, AcW118B
		Plan Chai Swamp	E103°39'09.7" N16°03'23.0"	7.8	32	AcWT41, AcWT82A, AcWT82B, AcW155
	Maha Sarakham	Irrigation canal	E103°19'08.4" N16°11'17.5"	7.6	31	AcW151
		Kosamphi Forest Park	E103°04'01.4" N16°15'14.6"	7.4	29	
	Khon Kaen	Bueng Kaen Nakhon Public Park	E102°50'07.4" N16°25'01.8"	7.7	31	AcWT14, AcW131A, AcW131B,
		Bung Nong Khot (Nong Khot Lake)	E102°47'58.7" N16°25'57.0"	7.7	31	AcW153A, AcW153B
		Pond, Nong Sa Pang	E102°49'59.3" N16°25'56.8"	7.9	31	
		Bueng Thung Sang	E102°51'21.9" N16°26'47.3"	7.6	32	
	Nakhon Ratchasima	Moat	E102°06'21.4" N14°58'13.1"	7.6	32	AcW111
		Chaloem Phra Kiat Phu Mirak Park	E102°06'04.7" N14°58'47.6"	7.8	31	AcWT29, AcW138
		Bung Ta Lua Park	E102°05'25.6" N14°57'45.3"	7.8	31	AcW30
West	Phetchaburi	Pond,City Pillar Shrine Phetchaburi (Old)	E99°57'10.3" N13°06'13.0"	7.6	33	AcW116
		Royal Thai Army 15th Military Circle Ramratcha Niwet Camp	E99°56'40.1" N13°05'36.0"	7.7	32	AcW117, AcW136, AcW137
	Ratchaburi	The Commemorating His Majesty The King's 60th Birthday Anniversary Park	E99°49'31.8" N13°32'17.8"	7.8	32	AcWT3, AcW125A, AcW125B
		Mahapathum Waterlily	E99°49'15.4" N13°48'52.3"	7.8	32	AcWT5, AcWT71, AcW126,

Region	Province	Place	GPS	pH	Temp (°C)	Isolate
	Kanchanaburi	Chaloem Phra Kiat Rama 9 Park	E99°30'37.4" N13°58'23.5"	7.7	30	AcW124
		Kanchanaburi College of Agriculture and Technology	E99°27'50.6" N13°59'28.3"	7.6	31	
	Suphan Buri	Fountain, Chao Por Lak Muang Suphan Shrine	E100°06'39.1" N14°28'43.0"	8.5	30	
		Bueng Chawak Chaloem Phrakiet	E100°02'50.3" N14°55'34.4"	7.6	29	AcW123
South	Prachuap Khiri Khan	Waterfall, Namtok Huai Yang National Park	E99°36'50.9" N11°37'31.4"	6.7	29	
		Ko Lak Park	E99°47'22.2" N11°48'58.3"	7.7	31	AcW152A, AcW152B
	Nakhon Si Thammarat	Princess Mother 84 pond (Thung Tha Lat)	E99°57'09.5" N8°27'00.9"	7.6	31	AcW115, AcW128
		Wang Si Lan Ska Waterfall	E99°44'58.3" N8°19'05.9"	6.2	29	
		Nan Chon Waterfall	E99°46'20.0" N8°18'19.0"	7.6	30	AcWT27, AcWT77, AcW113, AcW130
		Namtok Yong Waterfall	E99°44'30.6" N8°10'20.6"	6.3	29	AcWT9, AcW101, AcW108, AcW129
		Ta Pi River	E99°19'03.8" N9°08'19.1"	6.4	28.5	AcWT7, AcWT72, AcW127
	Surat Thani	Khun Thale Swamp	E99°19'52.4" N9°04'24.5"	7.6	30	
		Dat Fa Waterfall	E99°28'45.2" N8°51'48.6"	6.6	28	AcWT43, AcWT44, AcWT62, AcW119
	Songkhla	Chaloem Phra Kiat 80 Phansa Park	E100°34'52.8" N7°13'06.5"	7.5	30	AcWT86, AcW120, AcW142
		Hat Yai Park	E100°30'16.0" N7°02'32.6"	7.7	31	AcW36, AcW143
		Ton Nga Chang Waterfall	E100°14'05.4" N6°56'59.1"	6.5	28	
		Tone Ya Plong Waterfall	E100°31'31.4" N7°02'06.8"	6.6	29	
		Suan Tun Park	E100°37'02.6" N7°08'53.3"	7.6	29	AcW141
	Narathiwat	Bueng La Ha Swamp	E101°52'30.9" N5°54'46.4"	6.6	30	
		Juerae (Cherang) Swamp	E101°52'22.4" N5°54'34.3"	6.7	31	AcW114
		Namtok Sirindhorn Waterfall	E101°49'35.2" N5°48'09.4"	6.2	29	
		Sukirin	E101°44'27.9" N5°48'08.4"	6.9	31	
		Ang Kep Nam Ban Khok Yang Reservoir	E101°53'53.5" N 6°09'02.1"	6.5	30	AcW112A, AcW112B
		Cha Ro Canal	E101°59'52.3" N6°17'07.0"	6.6	30	AcWT37, AcWT39, AcWT79, AcWT81

Supplemental Table S2. Isolates with identical 18S rRNA gene sequences across > 2 kb. and their geographic origins.

Genotype	Allele	Isolates/GenBank Accession No.#	Origin*
T2/6B	I	AcWT1, AcW91	Bangkok, Chiangmai
	II	AcWT3, AcW159	Ratchaburi, Chiangmai
T3	I	AcW148, AcW150, AcW151, **S81337**	Chanthaburi, Buriram, Mahasarakham, **Scotland**
	II	AcWT27, AcWT93B, AcW139B, **MK124584, EF140628**	Nakhon Si Thammarat, Chiangmai, Lampang, **Russia, South Korea**
	III	AcWT20, AcWT23, AcW153B	Trat, Phitsanulok, Khon Kaen
T4A	I	AcWT65, AcWT82B,	Bangkok, Roi Et
T4B	I	AcWT71, AcWT72, AcWT79, AcW152, **GU808329**	Prachuap Khiri Khan, Nakhon Si Thammarat, Narathiwat, Ratchaburi, Bangkok
	II	AcWT63, AcW130B, AcW139A, AcW146, **GU808319, AF019058, AY026247, AY026249**	Ayuttaya, Nakhon Si Thammarat, Lampang, Chai Nat, Bangkok, **Brazil, Brazil, USA**
	III	AcWT82A, AcW131B	Roi Et, Khon Kaen
T4C	I	AcW113, AcW133, AcW134B, AcW145	Nakhon Si Thammarat, Bangkok, Ubon Ratchathani, Nakhon Sawan
	II	AcWT51, AcWT52	Phitsanulok, Nakhon Sawan
	III	AcW134A, AcW140A, U07409	Ubon Ratchathani, Chiangmai, **USA**
T4F	I	AcWT43, AcW135, AcW144, **GU808306**	Surat Thani, Trat, Chiangmai, Bangkok
	II	AcWT37, AcWT49	Narathiwat, Trat
T11	I	AcWT7, AcW160B	Nakhon Si Thammarat, Si Sa Ket

*Provinces in Thailand or country (bold).

Supplemental Table S3 Recombination in the 18S rRNA sequences within genotypes/subtypes and isolates of *Acanthamoeba.*

Genotype/ Subtype/ Isolate	Total Number of Sequences	Sequences with Recombination	Recombination Event Number	Breakpoint Positions#		Detection Methods, *P* Value						
				Begin	End	RDP	GENECONV	Bootscan	Maxchi	Chimaera	SiSscan	3Seq
**T2**	13	3										
			1	1172	1932	3.52×10^{-4}	3.52×10^{-4}	9.09×10^{-6}	1.69×10^{-7}	1.20×10^{-4}	2.03×10^{-8}	5.12×10^{-5}
			2	1391	2194	NS	5.73×10^{-4}	1.30×10^{-2}	4.76×10^{-5}	1.62×10^{-2}	8.34×10^{-6}	1.15×10^{-2}
			3	1158	1576	2.24×10^{-2}	2.95×10^{-3}	3.21×10^{-3}	2.28×10^{-2}	1.14×10^{-2}	7.75×10^{-4}	0.023817
**T2/6B**	8	1										
			1	1188	2075	NS	NS	NS	2.04×10^{-4}	4.08×10^{-4}	1.04×10^{-2}	1.36×10^{-4}
**T2/6C**	6	2										
			1	765	2127	NS	NS	NS	1.63×10^{-2}	NS	1.97×10^{-7}	NS
			2	1841	2238	NS	NS	2.63×10^{-2}	NS	NS	5.58×10^{-6}	3.95×10^{-4}
**T3**	25	1										
			1	672	1190	NS	NS	1.14×10^{-6}	1.64×10^{-8}	3.28×10^{-8}	3.52×10^{-6}	8.50×10^{-12}
**T4A**	78	20										
			1	1341	2097	NS	NS	3.32×10^{-2}	6.86×10^{-5}	2.75×10^{-4}	3.82×10^{-5}	2.11×10^{-3}
			2	1101	1329	NS	NS	NS	NS	NS	NS	5.04×10^{-4}
			3	848	1184	NS	NS	NS	NS	NS	3.47×10^{-3}	NS
			4	611	1944	NS	NS	NS	NS	NS	1.62×10^{-4}	3.27×10^{-2}
**T4B**	91	37										
			1	1691	2244	6.21×10^{-4}	3.98×10^{-3}	6.35×10^{-4}	9.22×10^{-6}	4.27×10^{-6}	NS	8.71×10^{-7}
			2	393	1207	NS	NS	NS	5.72×10^{-4}	NS	5.67×10^{-5}	8.71×10^{-7}
			3	906	2242	1.28×10^{-3}	2.25×10^{-3}	3.56×10^{-4}	1.86×10^{-6}	NS	3.31×10^{-14}	2.15×10^{-3}
			4	804	1310	NS	1.84×10^{-2}	1.21×10^{-3}	1.99×10^{-2}	3.19×10^{-2}	NS	4.56×10^{-6}
			5	506	1343	4.51×10^{-2}	NS	2.32×10^{-2}	7.23×10^{-4}	NS	NS	2.54×10^{-2}
			6	805	1343	NS	NS	NS	4.89×10^{-3}	NS	1.68×10^{-8}	2.24×10^{-3}
			7	1923	2244	NS	NS	NS	4.86×10^{-3}	NS	NS	3.22×10^{-3}
			8	1337	1710	NS	NS	3.55×10^{-2}	NS	NS	NS	NS

Genotype/ Subtype/ Isolate	Total Number of Sequences	Sequences with Recombination	Recombination Event Number	Breakpoint Positions#		Detection Methods, *P* Value						
				Begin	End	RDP	GENECONV	Bootscan	Maxchi	Chimaera	SiSscan	3Seq
**T4C**	53	17										
			1	568	2112	NS	4.70×10^{-5}	NS	1.24×10^{-3}	5.93×10^{-4}	4.87×10^{-15}	2.12×10^{-6}
			2	735	1418	NS	3.34×10^{-3}	NS	NS	NS	NS	3.45×10^{-5}
			3	767	1215	NS	NS	1.16×10^{-2}	NS	NS	2.13×10^{-2}	4.81×10^{-3}
			4	1025	1245	NS	6.74×10^{-3}	NS	NS	NS	NS	NS
			5	902	2090	NS	NS	NS	0.027	NS	NS	NS
			6	1333	2089	NS	NS	NS	3.17×10^{-2}	NS	NS	NS
**T4D**	28	5										
			1	938	1272	NS	1.34×10^{-4}	1.80×10^{-5}	5.32×10^{-7}	NS	NS	4.67×10^{-12}
			2	295	1580	NS	NS	NS	1.48×10^{-3}	NS	1.34×10^{-4}	NS
			3	1071	1580	NS	NS	NS	NS	NS	8.48×10^{-3}	NS
**T4E**	14	2										
			1	1388	2114	NS	NS	3.64×10^{-2}	7.17×10^{-4}	NS	3.50×10^{-2}	4.92×10^{-2}
**T4F**	40	4										
			1	1940	2218	NS	NS	NS	9.51×10^{-3}	3.18×10^{-2}	2.70×10^{-8}	5.21×10^{-8}
			2	1984	2239	NS	1.59×10^{-4}	1.45×10^{-3}	NS	NS	4.58×10^{-2}	1.23×10^{-3}
			3	1002	1215	NS	6.96×10^{-4}	7.54×10^{-4}	NS	NS	NS	0.045054
			4	2189	2288	NS	4.32×10^{-2}	NS	NS	NS	NS	NS
**T4G**	9	5										
			1	1266	2047	NS	NS	4.17×10^{-2}	0.0133	NS	NS	NS
			2	747	995	NS	NS	NS	1.74×10^{-2}	NS	NS	NS
**T5**	31	18										
			1	1486	2279	2.98×10^{-12}	9.01×10^{-24}	6.69×10^{-20}	1.61×10^{-2}	8.93×10^{-3}	NS	NS
			2	51	1541	6.35×10^{-7}	9.51×10^{-18}	2.25×10^{-17}	4.15×10^{-9}	7.22×10^{-5}	1.72×10^{-55}	4.19×10^{-12}
			3	205	289	9.09×10^{-3}	1.70×10^{-4}	NS	9.43×10^{-4}	8.51×10^{-4}	NS	NS
			4	176	288	NS	2.34×10^{-3}	NS	NS	NS	8.68×10^{-6}	NS
			5	1418	1540	NS	NS	2.54×10^{-2}	2.40×10^{-2}	2.25×10^{-2}	4.75×10^{-5}	6.62×10^{-3}

Genotype/ Subtype/ Isolate	Total Number of Sequences	Sequences with Recombination	Recombination Event Number	Breakpoint Positions#		Detection Methods, *P* Value						
				Begin	End	RDP	GENECONV	Bootscan	Maxchi	Chimaera	SiSscan	3Seq
**T6**	3	1										
			1	139	1698	NS	NS	NS	4.44×10^{-2}	NS	NS	NS
**T9**	9	3										
			1	87	1734	NS	5.57×10^{-4}	6.51×10^{-7}	1.17×10^{-2}	1.12×10^{-2}	NS	2.37×10^{-5}
			2	206	1184	NS	2.65×10^{-2}	NS	1.19×10^{-4}	NS	9.84×10^{-21}	3.29×10^{-6}
			3	795	2222	NS	NS	NS	0.01609	4.33×10^{-3}	NS	NS
**T11**	19	6										
			1	1095	1234	NS	1.76×10^{-8}	3.87×10^{-8}	2.75×10^{-2}	0.02584	NS	2.68×10^{-5}
			2	735	983	3.04×10^{-3}	NS	0.03246	8.77×10^{-7}	4.09×10^{-4}	NS	2.30×10^{-4}
			3	2065	2200	NS	6.96×10^{-4}	1.60×10^{-2}	NS	NS	NS	NS
			4	279	1887	NS	NS	NS	NS	7.67×10^{-3}	NS	NS
			5	1334	1953	NS	NS	NS	4.99×10^{-2}	NS	2.02×10^{-9}	NS
			6	1341	1935	NS	NS	NS	9.25×10^{-3}	NS	NS	NS
**T12**	4	1										
			1	359	2204	NS	5.13×10^{-8}	1.25×10^{-5}	3.45×10^{-3}	1.56×10^{-3}	3.97×10^{-4}	6.98×10^{-7}
**T13**	4	3										
			1	86	2214	NS	NS	NS	4.38×10^{-2}	NS	4.62×10^{-16}	1.57×10^{-5}
			2	1412	1734	NS	NS	4.51×10^{-3}	8.72×10^{-3}	1.29×10^{-3}	1.06×10^{-4}	NS
			3	793	884	1.40×10^{-3}	1.90×10^{-2}	9.34×10^{-4}	4.65×10^{-2}	3.40×10^{-2}	5.50×10^{-3}	NS
**T15**	10	3										
			1	344	741	NS	1.21×10^{-3}	2.85×10^{-3}	1.81×10^{-6}	8.94×10^{-4}	NS	2.16×10^{-2}
			2	342	714	NS	NS	4.33×10^{-2}	2.43×10^{-4}	4.92×10^{-2}	3.43×10^{-11}	4.35×10^{-3}
**T16**	3	2										
			1	70	2087	NS	NS	NS	NS	NS	NS	3.48×10^{-2}
**T17**	7	3										
			1	60	1493	NS	9.30×10^{-3}	1.97×10^{-2}	2.87×10^{-4}	8.59×10^{-4}	4.38×10^{-9}	3.80×10^{-5}
			2	1348	1551	NS	3.13×10^{-2}	NS	NS	NS	NS	NS

Genotype/ Subtype/ Isolate	Total Number of Sequences	Sequences with Recombination	Recombination Event Number	Breakpoint Positions#		Detection Methods, *P* Value						
				Begin	End	RDP	GENECONV	Bootscan	Maxchi	Chimaera	SiSscan	3Seq
**T18**	18	5										
			1	1499	2221	NS	5.93×10^{-4}	4.79×10^{-4}	3.68×10^{-6}	8.02×10^{-4}	1.02×10^{-13}	NS
			2	732	1306	NS	NS	NS	8.43×10^{-6}	1.02×10^{-2}	NS	6.90×10^{-3}
			3	880	1302	3.14×10^{-2}	1.13×10^{-2}	1.40×10^{-3}	1.90×10^{-4}	9.20×10^{-5}	9.14×10^{-6}	2.69×10^{-2}
			4	832	2066	NS	NS	NS	0.04252	2.48×10^{-4}	1.27×10^{-7}	4.16×10^{-2}
			5	220	1498	3.50×10^{-2}	NS	3.23×10^{-2}	NS	NS	2.85×10^{-2}	5.67×10^{-4}
**T20**	12	2										
			1	384	801	NS	1.60×10^{-17}	NS	9.32×10^{-18}	NS	NS	2.27×10^{-38}
			2	885	2088	2.28×10^{-4}	1.45×10^{-3}	1.06×10^{-4}	2.08×10^{-10}	4.07×10^{-2}	2.17×10^{-9}	1.63×10^{-2}
**E1**	3	1										
			1	1493	2231	NS	4.11×10^{-4}	3.02×10^{-4}	6.50×10^{-5}	9.24×10^{-4}	NS	6.09×10^{-8}
**E2**	3	1										
			1	1731	2175	NS	2.13×10^{-3}	7.12×10^{-3}	NS	NS	NS	1.36×10^{-2}
**E12**	4	2										
			1	256	1823	NS	NS	1.18×10^{-3}	NS	NS	NS	6.94×10^{-3}
**F1**	5	2										
			1	691	1331	NS	NS	NS	8.56×10^{-3}	NS	2.45×10^{-4}	NS
**P12**	5	1										
			1	1038	2115	NS	NS	NS	NS	NS	NS	4.25×10^{-2}

Relative to the 18S rRNA sequence of *A. castellanii* (GenBank accession no. U07413).
NS denotes non-significant *p* value.

Supplemental Table S4. Percentages of sequence identity between AcW61A and representative genotypes including AcW61B.

Comparison with genotype (GenBank Accession No.)	Aligned sites	Identical sites	%Identity
T1 (U07400)	2251	2075	92.18
T2 (U07411)	2266	2002	88.35
T2/6A (AY026244)	2272	1993	87.72
T2/6B (AB425945)	2270	1999	88.06
T2/6C (AF019051)	2269	1996	87.97
T3 (U07412)	2276	2035	89.41
T4A (U07413)	2275	2049	90.07
T4B (U07414)	2265	2032	89.71
T4C (U07409)	2276	2047	89.94
T4D (AY351644)	2276	2048	89.98
T4E (AF019061)	2275	2035	89.45
T4F (AF316547)	2270	2044	90.04
T4G (U07416)	2284	2036	89.14
T5 (U94739)	2360	1958	82.97
T6 (AF019063)	2269	1977	87.13
T7 (AF019064)	2672	1912	71.56
T8 (AF019065)	2521	1884	74.73
T9 (AF019066)	2565	1893	73.80
T10 (AF019067)	2285	2031	88.88
T11 (AF019068)	2271	2045	90.05
T12 (AF019070)	2310	2013	87.14
T13 (AF132134)	2270	2028	89.34
T14 (AF333609)	2302	2101	91.27
T15 (KY513791)	2318	2038	87.92
T16 (AY026245)	2246	2044	91.01
T17 (GU808277)	2579	1897	73.56
T18 (KC822461)	2581	1905	73.81
T19 (KJ413084)	2306	2074	89.94
T20 (DQ451161)	2245	2054	91.49
T21 (KX840327)	2268	1909	84.17
T22 (CDEZ01000000)	2155	1871	86.82
T23 (AcW61B)	2244	2221	98.98

Supplemental Table S5. *Acanthamoeba* 18S rRNA sequences from public database and their geographic origins.

GenBank Accession No	Geographic Origin	No. Nucleotides*
AB425945	Italy	2246
AB425948	Italy	2249
AB425949	Italy	2252
AB425952	Italy	2229
AB425955	Italy	2246
AF005995	South Korea	2292
AF005996	South Korea	2294
AF005997	South Korea	2285
AF005998	South Korea	2292
AF019050	France	2238
AF019051	USA	2239
AF019052	USA	2239
AF019053	USA	2249
AF019054	UK	2249
AF019055	China	2246
AF019056	Mexico	2237
AF019057	USA	2245
AF019058	USA	2244
AF019059	USA	2256
AF019060	USA	2224
AF019061	USA	2253
AF019062	UK	2243
AF019063	France	2249
AF019064	USA	2682
AF019065	USA	2517
AF019066	France	2572
AF019067	USA	2292
AF019068	USA	2242
AF019069	USA	2236

GenBank Accession No	Geographic Origin	No. Nucleotides*
AF019070	Barbados	2323
AF114438	Austria	2301
AF132134	Germany	2257
AF132135	Germany	2233
AF132136	Germany	2253
AF251937	Austria	2242
AF251938	Austria	2245
AF251939	Austria	2244
AF260718	Germany	2235
AF260719	France	2244
AF260720	Austria	2242
AF260721	Austria	2231
AF260722	Austria	2252
AF260723	Austria	2228
AF260724	Austria	2239
AF260725	Austria	2245
AF285277	Austria	2243
AF316545	South Korea	2293
AF316546	South Korea	2333
AF316547	South Korea	2308
AF333607	Pakistan	2377
AF333608	Pakistan	2415
AF333609	Pakistan	2377
AF346662	USA	2181
AF349044	South Korea	2298
AF349045	South Korea	3242
AF352387	USA	2235
AF352388	USA	2235
AF352389	Malaysia	2259
AF352390	USA	2255
AF352391	Malaysia	2990

GenBank Accession No	Geographic Origin	No. Nucleotides*
AF366580	Malaysia	2251
AM408796	Austria	2287
AM408799	Austria	2277
AM408800	Tunesia	2280
AM408801	Austria	2290
AM408802	Austria	2283
AM408803	Austria	2230
AM941721	Austria	2224
AY026242	USA	2559
AY026243	USA	2239
AY026244	USA	2244
AY026245	Brazil	2217
AY026246	Brazil	2239
AY026247	Brazil	2233
AY026248	Brazil	2225
AY026249	Brazil	2239
AY026250	Brazil	2237
AY026251	Brazil	2243
AY026747	Brazil	2244
AY026748	Brazil	2237
AY026749	Brazil	2243
AY148954	South Korea	3236
AY148955	South Korea	2294
AY148956	South Korea	2286
AY148957	South Korea	2288
AY148958	South Korea	2283
AY148963	South Korea	2287
AY148964	South Korea	2295
AY172999	South Korea	2314
AY173000	South Korea	2310
AY173001	South Korea	2322

GenBank Accession No	Geographic Origin	No. Nucleotides*
AY173002	South Korea	2312
AY173003	South Korea	2300
AY173004	South Korea	2311
AY173005	South Korea	2314
AY173006	South Korea	2370
AY173009	South Korea	2314
AY173010	South Korea	2315
AY173011	South Korea	2314
AY173013	South Korea	2375
AY173014	South Korea	3256
AY173015	South Korea	3275
AY176047	South Korea	3583
AY262360	USA	1443
AY351644	UK	2243
AY351645	France	2227
AY351646	France	2247
AY351647	Morocco	2224
AY552093	Canada	2141
AY552094	Canada	2141
AY552095	Canada	2141
AY552096	Canada	2141
AY694138	India	2250
AY694139	India	2245
AY694140	India	2245
AY694141	India	2241
AY694142	India	2193
AY694143	India	2250
AY694144	India	2241
AY702983	USA	2245
AY703004	USA	2237
AY703024	USA	2237

GenBank Accession No	Geographic Origin	No. Nucleotides*
CDEZ010000000	USA	24098
DQ103890	Austria	2241
DQ185605	Turkey	2582
DQ185606	Turkey	2249
DQ185607	Turkey	2579
DQ264391	Turkey	2255
DQ451161	USA	2244
DQ451163	USA	2238
DQ499151	USA	1795
DQ992178	Egypt	2666
DQ992179	Egypt	2247
DQ992180	Egypt	2247
DQ992181	Egypt	2246
DQ992182	Egypt	2249
DQ992183	Egypt	2249
DQ992184	Egypt	2181
DQ992185	Egypt	2255
DQ992186	Egypt	2302
DQ992187	Egypt	2302
DQ992188	Egypt	2302
DQ992189	Egypt	2302
DQ992190	Egypt	2304
DQ992192	Egypt	2236
DQ992193	Egypt	2320
EF140625	South Korea	2287
EF140626	South Korea	2280
EF140627	South Korea	2276
EF140628	South Korea	2284
EF140629	South Korea	2286
EF140631	South Korea	2250
EF140632	South Korea	2242

GenBank Accession No	Geographic Origin	No. Nucleotides*
EF140633	South Korea	3262
EF140638	South Korea	2285
EF140639	South Korea	2292
EF205325	Spain	2227
EF378672	USA	1538
EF429130	Austria	2243
EF429131	Austria	2248
EF554328	USA	2236
EU683884	Spain	2222
EU686716	Spain	2262
FJ807647	USA	2274
GQ397468	Slovakia	1976
GQ397476	Slovakia	2395
GQ889265	USA	2241
GQ905495	Slovakia	1520
GQ905499	Czech Republic	1974
GQ924681	USA	2275
GQ924682	USA	2274
GU001160	UK	7843
GU320583	USA	2271
GU320589	USA	2272
GU808277	Thailand	2581
GU808278	Thailand	2556
GU808279	Thailand	2570
GU808280	Thailand	2569
GU808281	Thailand	2572
GU808282	Thailand	2559
GU808283	Thailand	2235
GU808284	Thailand	2240
GU808285	Thailand	2236
GU808286	Thailand	2238

GenBank Accession No	Geographic Origin	No. Nucleotides*
GU808287	Thailand	2240
GU808288	Thailand	2240
GU808289	Thailand	2246
GU808290	Thailand	2245
GU808291	Thailand	2173
GU808292	Thailand	2228
GU808293	Thailand	2228
GU808294	Thailand	2230
GU808295	Thailand	2228
GU808296	Thailand	2236
GU808297	Thailand	2235
GU808298	Thailand	2231
GU808299	Thailand	2228
GU808300	Thailand	2228
GU808301	Thailand	2574
GU808302	Thailand	2574
GU808303	Thailand	2236
GU808304	Thailand	2240
GU808305	Thailand	2236
GU808306	Thailand	2228
GU808307	Thailand	2228
GU808308	Thailand	2236
GU808309	Thailand	2236
GU808310	Thailand	2156
GU808311	Thailand	2242
GU808312	Thailand	2243
GU808313	Thailand	2248
GU808314	Thailand	2247
GU808315	Thailand	2248
GU808316	Thailand	2243
GU808317	Thailand	2244

**GenBank Accession No**	**Geographic Origin**	**No. Nucleotides***
GU808318	Thailand	2231
GU808319	Thailand	2236
GU808320	Thailand	2280
GU808321	Thailand	2239
GU808322	Thailand	2238
GU808323	Thailand	2240
GU808324	Thailand	2240
GU808325	Thailand	2241
GU808326	Thailand	2236
GU808327	Thailand	2236
GU808328	Thailand	2236
GU808329	Thailand	2236
HM159368	Austria	2236
HM363628	Germany	2286
JQ271663	Czech Republic	2274
JQ271664	Czech Republic	2285
JQ271665	Czech Republic	2284
JQ271666	Czech Republic	2282
JQ271667	Czech Republic	2284
JQ669661	USA	2256
JX423610	USA	2251
JX423611	Mexico	2271
KC822461	USA	2633
KC822462	USA	2629
KC822463	USA	2629
KC822464	USA	2629
KC822465	USA	2629
KC822466	USA	2639
KC822467	USA	2629
KC822468	USA	2628
KC822469	USA	2630

GenBank Accession No	Geographic Origin	No. Nucleotides*
KC822470	USA	2628
KF318460	Brazil	2265
KF318462	UK	2256
KF433820	Equatorial Guinea	2116
KF433822	Equatorial Guinea	2145
KF433824	Equatorial Guinea	2188
KF928933	Netherlands	1869
KF928948	Italy	1503
KF928950	China Tibet	2198
KJ413084	Spain	2301
KP711387	Brazil	2288
KR780546	Poland	2172
KR780547	Poland	2172
KR780548	Poland	2170
KR780549	Poland	2170
KR780550	Poland	2170
KR780551	Poland	2171
KR780552	Poland	2171
KR780553	Poland	2171
KR780554	Poland	2171
KR780555	Poland	2171
KR780556	Poland	2171
KR780557	Poland	2178
KR780558	Poland	2178
KR780559	Poland	2119
KR780560	Poland	2130
KR780562	Poland	2130
KR780563	Poland	2131
KR780564	Poland	2130
KR780565	Poland	2130
KR780566	Poland	2130

GenBank Accession No	Geographic Origin	No. Nucleotides*
KR780567	Poland	2130
KR780568	Poland	2130
KR780569	Poland	2129
KX069000	Thailand	1829
KX840327	China Tibet	2248
KY072778	Spain	2068
KY072779	Spain	2118
KY513789	France	2256
KY513790	France	2264
KY513791	France	2327
KY513792	France	2338
KY513793	France	2257
KY513794	France	2353
KY513795	France	2290
KY827389	China	2255
KY827390	China	2252
KY827391	China	2292
KY827392	China	2252
KY934458	France	2198
M13435	USA	2303
MF113385	Spain	2143
MF350344	Egypt	2297
MF350345	Egypt	2295
MF350346	Egypt	2950
MF350347	Egypt	2812
MG825484	Brazil	1644
MH087092	Spain	2129
MH087093	Spain	2079
MK124583	Russia	2261
MK124584	Russia	2288
MK124585	Russia	2262

GenBank Accession No	Geographic Origin	No. Nucleotides*
MK124586	Russia	2261
MK124587	Russia	2265
MK124588	Russia	2275
MK248592	Brazil	3449
MN153011	UK	2239
MN153013	Austria	2247
MN153015	Austria	2230
MN153016	Austria	2240
MN153017	Austria	2243
MN153018	Austria	2237
MN153019	Austria	2292
MN153020	Austria	2291
MN153021	Austria	2294
MN153022	Austria	2246
MN153024	Austria	2331
MN153025	Austria	2255
MN153026	Austria	2306
MN153027	Austria	2252
MN153028	Austria	2583
MN239984	USA	2331
MN239992	UK	2610
S81337	UK	2781
U07400	USA	2282
U07401	India	2287
U07402	USA	2273
U07403	USA	2282
U07404	USA	2290
U07405	USA	2284
U07406	USA	2280
U07407	USA	2287
U07408	USA	2293

GenBank Accession No	Geographic Origin	No. Nucleotides*
U07409	USA	2298
U07410	USA	2282
U07411	Israel	2288
U07412	USA	2812
U07413	UK	2290
U07414	USA	2278
U07415	Japan	2276
U07416	USA	2299
U07417	UK	2278
U94730	Germany	2291
U94731	France	2292
U94732	Germany	2291
U94733	USA	2293
U94734	USA	3012
U94735	USA	2935
U94736	Germany	2934
U94737	USA	2930
U94738	USA	2936
U94739	USA	2992
U94740	Germany	2973
U94741	France	2950

* Sequences > 1.4 kb are included for some rare genotypes.

Supplemental Figure S1. Variation in intron of genotype T3. Indels located in short repeat regions are shown in bold and marked with vertical red lines underneath. Asterisks indicate identical residues. The proposed helices shown as P1-P9 and cP1-cP9 are highlighted and indicated above the alignment.

```
               P1            cP1                 P2                                    cP2
Allele I       UAACAUAUUAUCAGCCAGAUGUUGAUAUCAUACUACACACACAACAGAGUACUACUAGUGCUGCUCUCUUGUUGUUGUGG
Allele II      UAACAUAUUAUCAGCCAGAUGUUGAUAUCAUACUACACACACAACAGAGUACUACUAGUGCUGCUCUCUUGUUGUUGUGG
Allele III     UAACAUA---GCAGCCAGAUGUUGAUAUCAUACAAUGCAUGAGAGAGAGUACUAUUAG---UGCUCUCUUGUU---GU-G
Allele IV      UAACAUA---GCAGCCAGAUGUUGAUAUCAUACAAUGCAUGAGAGAGAGUACUAUUAG---UGCUCUCUUGUU---GU-G
Allele V       UAACAUA---GCAGCCAGAUGUUGAUAUCAUACA---------ACAGAGUACUACUAG---UGCUCUCUUGGUGU-GUGU
               *******    **********************          * ********* ***   ********** *   **
                                                  ||||||||                         ||||||||||

                                    P2.1          cP2.1      P3        P4       P5       P5.2
Allele I       GUGUGUGAUAUUGGCAGAAAGUUGGAGAUAAGCACCAACUGUACAACCGUAAAUUGCGGGAAACCCUAAAGCCUAUUCU
Allele II      GUGUGUGAUAUUGGCAGAAAGUUGGAGAUAAGCACCAACUGUAC-ACCGUAAAUUGCGGGAAACCCUAAAGCCUAUUCU
Allele III     GUGUGUGAUAUUGGCAGAAAGUUGGAGAUAAGCACCAACUGUACAACCGUAAAUUGCGGGAAACCCUAAAGCCUAUUCU
Allele IV      GUGUGUGAUAUUGGCAGAAAGUUGGAGAUAAGCACCAACUGUACAACCGUAAAUUGCGGGAAACCCUAAAGCCUAUUCU
Allele V       GUGUGUGAUAUUGGCAGAAAGUUGGAGAUAAGCACCAACUGUACAACCGUAAAUUGCGGGAAACCCUAAAGCCUAUUCU
               ******************************************** ***********************************

                      P5.3           cP5.3        5.4         cP5.4              cP5.2    P5.5
Allele I       ACCGCGGCUAUGAGGAUGACUCAUAGUGCAGCAGGUGUGGAUAACGCCCACCGGAUGGUAAUAACGAAUAGGAUGACCUU
Allele II      ACCGCGGCUAUGAGGAUGACUCAUAGUGCAGCAGGUGUGGAUAACGCCCACCGGAUGGUAAUAACGAAUAGGAUGACCUU
Allele III     ACCGCGGCUAUGAGGAUGACUCAUAGUGCAGCAGGUGCGGAUAACGCCCACCGGAUGGUAAUAACGAAUAGGAUGACCUU
Allele IV      ACCGCGGCUAUGAGGAUGACUCAUAGUGCAGCAGGUGCGGAUAACGCCCACCGGAUGGUAAUAACGAAUAGGAUGACCUU
Allele V       ACCGCGGCUAUGAGGAUGACUCAUAGUGCAGCAGGUGUGGAUAACGCCCACCGGAUGGUAAUAACGAAUAGGAUGACCUU
               ************************************* ******************************************

                                             cP5.5    cP5    cP4   P6    P6a        cP6a   cP6
Allele I       AGUACCUCCAUAA-CAUAAUAAUAACAAAAGGUCGCAAUGGGCAAUCCGCAGCCAAGCUCCCAUAUGCUGGGAGAAGGUU
Allele II      AGUACCUCCAUAAGCAUAAUAAUAACAAAAGGUCGCAAUGGGCAAUCCGCAGCCAAGCUCCCAUAUGCUGGGAGAAGGUU
Allele III     AGUACCUCCACA--UAACAUAAUAACAAAAGGUCACAAUGGGCAAUCCGCAGCCAAGCUCCCAUAUGUUGGGAGAAGGUU
Allele IV      AGUACCUCCACA--UAACAUAAUAACAAAAGGUCACAAUGGGCAAUCCGCAGCCAAGCUCCCAUAUGUUGGGAGAAGGUU
Allele V       AGUACCUCCAUA-----CAUAACACAAAAAGGUCACAAUGGGCAAUCCGCAGCCAAGCUCCCAUAUGUUGGGAGAAGGUU
               ********** *      **** *  ******** ******************************** ************

                    P7      cP3   P8      cP8       cP7          P9       cP9      P9.1
Allele I       CAGAGACUAUAAUCGGAUGGGCGCAAGCUUAAGGUAUAGUCCACUCCCACUUCGAAAGAGGUGCUAUAAAACUAUUAAGA
Allele II      CAGAGACUAUAAUCGGAUGGGCGCAAGCUUAAGGUAUAGUCCACUCCCACUUCGAAAGAGGUGCUAUAAAACUAUUAAGA
Allele III     CAGAGACUAUAAUCGGAUGGGCGCAAGCUUAAGGUAUAGUCCACUCCCACUUCGAAAGAGGUGCUAUAAAGCUAUUAAGA
Allele IV      CAGAGACUAUAAUCGGAUGGGCGCAAGCUUAAGGUAUAGUCCACUCCCACUUCGAAAGAGGUGCUAUAAAGCUAUUAAGA
Allele V       CAGAGACUAUAAUCGGAUGGGCGCAAGCUUAAGGUAUAGUCCACUCCCACUUCGAAAGAGGUGCUAUAAAGCUAUUAAGA
               ********************************************************************** *********

                              cP9.1             P9.2             cP9.2           P9.3
Allele I       AGUUUGCUUACUUCCUAGGGUUUUAUAGAUCCGUUGCUUGUAUAUAUACAACAUACGUGAGUGUGUGGUGUAUACAUCAA
Allele II      AGUUUGCUUACUUCCUAGGGUUUUAUAGAUCCGUUGCUUGUAUAUAUACAACAUACGUGAGUGUGUGGUGUAUACAUCAA
Allele III     AGUUUGCUUACUUCCUAGGGUUUUAUAGAUCCGUUGCUUGUA----UACAAUAUACG-----------UGUAUACAUCAA
Allele IV      AGUUUGCUUACUUCCUAGGGUUUUAUAGAUCCGUUGCUUGGA----UACAAUAUACG-----------UGUAUACAUCAA
Allele V       AGUUUGCUUACUUCCUAGGGUUUUAUAGAUCCGUUGCUUGUA----UACA-UA----------------GUAUACAUCAA
               **************************************** *    ****  **               ***********
                                                        ||||||         |||||||||||||||

              cP9.3  P9.4        cP9.4
Allele I       UACCCCCAAGAGGAAAUGCUUGGUUGGGAAGGAUGGUAGCAAGG
Allele II      UACCCCCAAG--GAAAUGCUUGGUUG-GAA-GAUGGUAGCAAGG
Allele III     UACC-CCAAGAGGAAAUGCUUGGUUG-GAAGGAUGGUAGCAAGG
Allele IV      UACC-CCAAGAGGAAAUGCUUGGUUG-GAAGGAUGGUAGCAAGG
Allele V       UACC-CCAAGAGGAAAUGCUUGGUUG-GAAGGAUGGUAGCAAGG
               **** ***** * *************    * *************
                 |||||
```

Allele I: GQ397468, GQ905499 and AcWT29
Allele II: MF350347 and U07412
Allele III: S81337, AcWT20, AcWT23, AcWT27, AcWT93B, AcW105, AcW112B, AcW139B, AcW147, AcW148, AcW149, AcW150 and AcW151
Allele IV: AcW153B
Allele V: AcW109B

Supplemental Figure S2. Secondary structure of intron in genotype T3. Regions with nucleotide substitution, insertion and deletion from Supplemental Figure S1are highlighted in yellow, green and blue, respectively. Predicted intron structure model after reference 27.

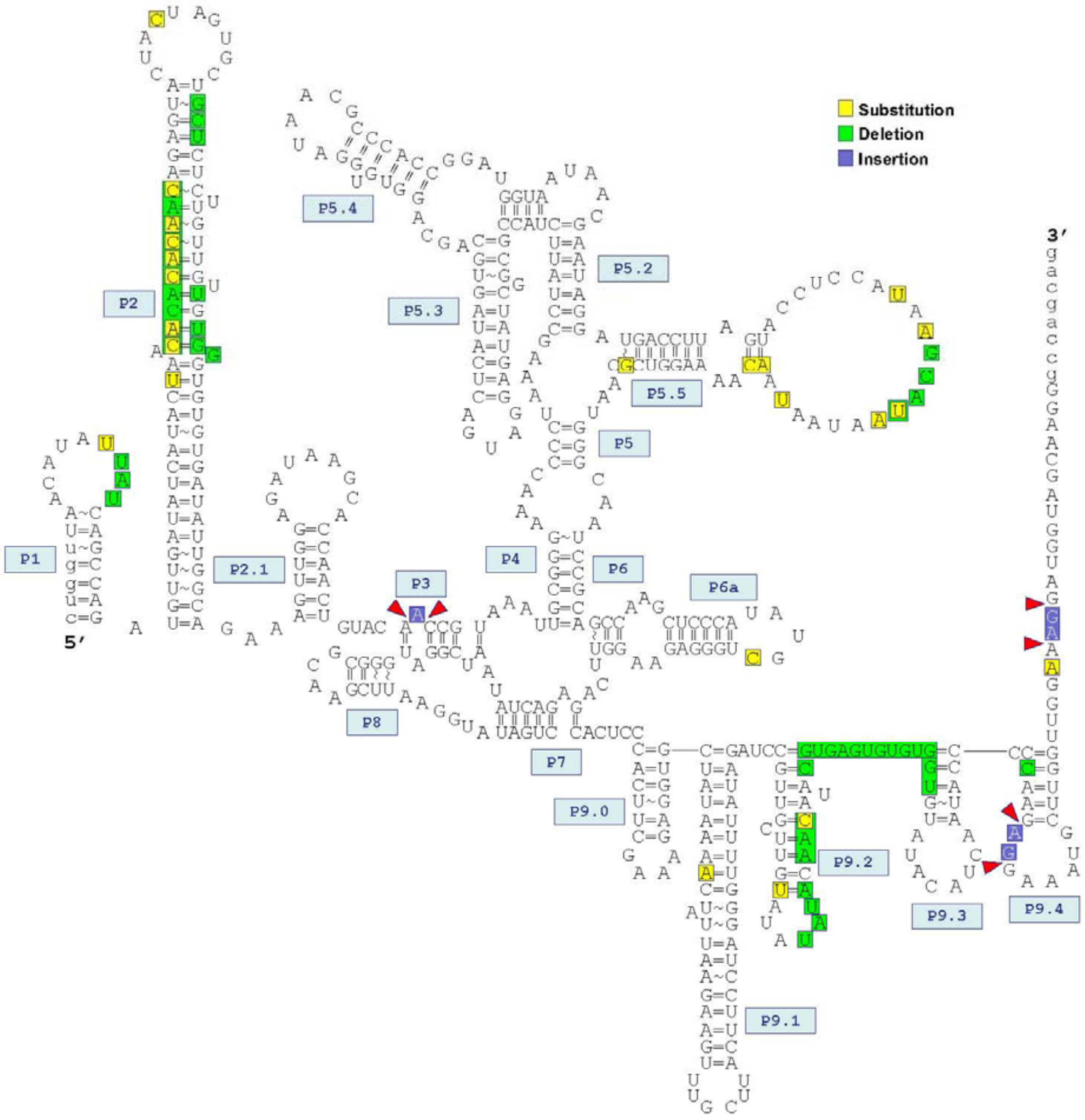

Supplemental Figure S3. Maximum likelihood tree inferred from the 18S rRNA sequences of representative genotypes and subtypes of *Acanthamoeba.* The tree has been constructed in MEGA 6 program based on the Tamura-Nei model using a discrete Gamma distribution model for rate differences among sites [59]. Bootstrap values > 50% from 1,000 pseudo-resamplings are shown along the branches. Scale represent number of nucleotide substitution per site. The novel genotype 'T23' are marked with filled red circles. All sequences are > 2kb.

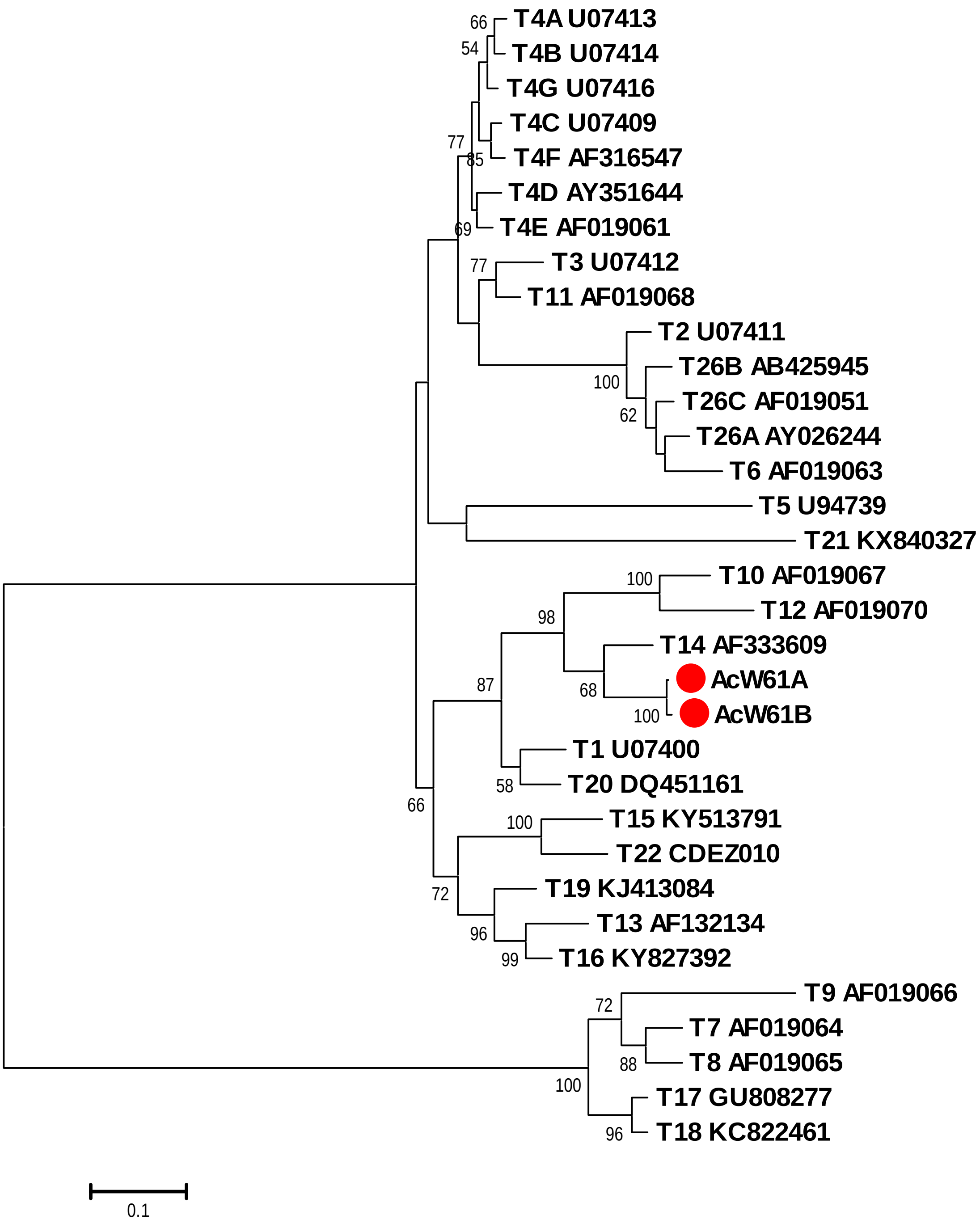

Supplemental Figure S4. Maximum likelihood tree inferred from sequences of genotype T11, representative genotype T3 and subtypes T4A-T4G. Bootstrap values from 1,000 pseudoreplicates are shown along the branches. Scale denotes number of nucleotide substitution per site.

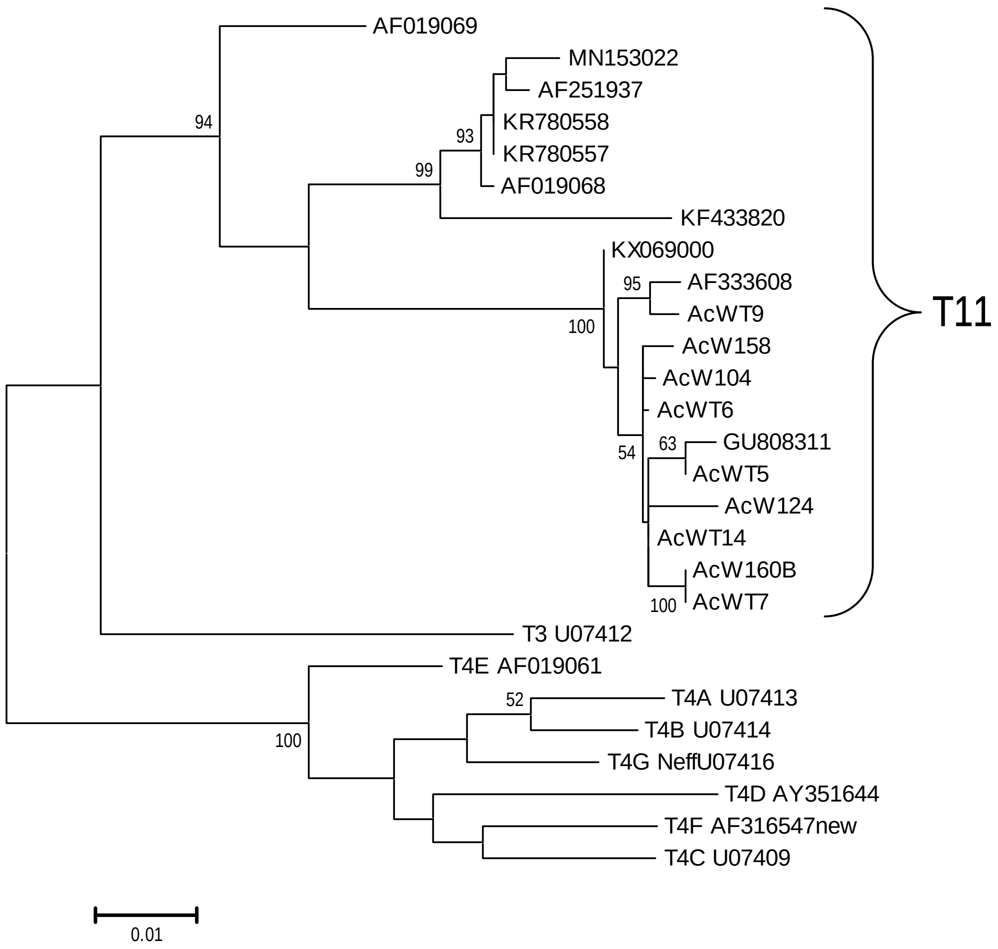

Supplement: Supplementary file 1 — Supplementary Information. [file 41598_2021_96690_MOESM1_ESM.pdf]
